# Evaluation of stenoses using AI video models applied to coronary angiography

**DOI:** 10.1038/s41746-024-01134-4

**Published:** 2024-05-23

**Authors:** Élodie Labrecque Langlais, Denis Corbin, Olivier Tastet, Ahmad Hayek, Gemina Doolub, Sebastián Mrad, Jean-Claude Tardif, Jean-François Tanguay, Guillaume Marquis-Gravel, Geoffrey H. Tison, Samuel Kadoury, William Le, Richard Gallo, Frederic Lesage, Robert Avram

**Affiliations:** 1 Department of Electrical Engineering, Polytechnique Montréal, Montreal, QC Canada; 2 Heartwise (heartwise.ai), Montreal Heart Institute, Montreal, QC Canada; 3 Department of Medicine, Montreal Heart Institute, Université de Montréal, Montreal, QC Canada; 4 Department of Medicine, University of California, San Francisco, CA USA; 5 Department of Computer Engineering, Polytechnique Montréal, Montreal, QC Canada

**Keywords:** Cardiovascular diseases, Machine learning, Image processing

## Abstract

The coronary angiogram is the gold standard for evaluating the severity of coronary artery disease stenoses. Presently, the assessment is conducted visually by cardiologists, a method that lacks standardization. This study introduces DeepCoro, a ground-breaking AI-driven pipeline that integrates advanced vessel tracking and a video-based Swin3D model that was trained and validated on a dataset comprised of 182,418 coronary angiography videos spanning 5 years. DeepCoro achieved a notable precision of 71.89% in identifying coronary artery segments and demonstrated a mean absolute error of 20.15% (95% CI: 19.88–20.40) and a classification AUROC of 0.8294 (95% CI: 0.8215–0.8373) in stenosis percentage prediction compared to traditional cardiologist assessments. When compared to two expert interventional cardiologists, DeepCoro achieved lower variability than the clinical reports (19.09%; 95% CI: 18.55–19.58 vs 21.00%; 95% CI: 20.20–21.76, respectively). In addition, DeepCoro can be fine-tuned to a different modality type. When fine-tuned on quantitative coronary angiography assessments, DeepCoro attained an even lower mean absolute error of 7.75% (95% CI: 7.37–8.07), underscoring the reduced variability inherent to this method. This study establishes DeepCoro as an innovative video-based, adaptable tool in coronary artery disease analysis, significantly enhancing the precision and reliability of stenosis assessment.

## Introduction

Cardiovascular diseases account for roughly 17.9 million annual deaths, making them the leading global cause of mortality^1^. A significant contributor is atherosclerotic coronary artery disease (CAD), where stenoses (i.e. obstructions caused by atherosclerotic plaque) can lead to myocardial infarction if untreated^2–4^. Reliable and accurate identification of the extent and severity of CAD directly impacts the decision to pursue an invasive revascularization procedure (i.e. percutaneous coronary intervention (PCI), or coronary artery bypass grafting (CABG)), generally conducted when stenoses are severe^2^. In addition, this stenosis assessment is essential to provide necessary treatment and prevent unnecessary revascularization^5^. These stenoses are usually identified through visual interpretation of coronary angiography (CAG) videos, a minimally invasive procedure involving iodine dye and X-ray imaging^4,6–8^. Despite the routine use of visual estimation of stenosis in CAG, this approach lacks standardization and shows high intra-observer and inter-observer variability, generally ranging from 6.9% to 26.4% between observers^9^. Yet, this visual assessment remains the clinical standard for assessing CAD severity^4,10,11^ and is also endorsed by clinical guidelines^12,13^. Quantitative coronary angiography (QCA) offers more reproducible results but requires clinician input for image selection and is resource-intensive, thus leading to its predominant use in research^5,14–16^. Adjunctive testing, such as physiological assessments (e.g. fractional flow reserve; FFR)^17^ and intra-vascular imaging^18^, supplements CAD evaluation during coronary angiography. These methods, however, are utilized in a minority of cases (10–20% of coronary angiograms^17–19^) due to the need for additional expertise and invasive equipment. Furthermore, the primary determination for adjunctive testing relies on the cardiologist’s visual estimation of angiograms to identify intermediate-severity or greater stenoses (e.g. 40–69%). The SYNTAX score, assessing CAD severity, guides revascularization decisions between CABG or PCI based on stenosis location, calcification, and length, obtained through visual assessment^20^. Additionally, metrics like the thrombolysis in myocardial infarction flow grading and myocardial blush grading evaluate the impact of stenosis on epicardial and microvascular circulation, respectively^20^. Despite these tools, the primary method for assessing stenosis severity is the cardiologist’s visual examination of CAG videos, highlighting the need for an efficient, objective assessment tool in clinical practice.

Artificial intelligence (AI) algorithms offer the potential for more standardized assessments of diagnostic tests, such as CAG, often performing as well or better than medical experts in various tasks^1^. However, existing AI methods for the interpretation of CAG face several challenges that hinder their clinical implementation: they often were trained on small datasets^3,7,21^, have extensive exclusion criteria^3,14,22^, rely on classifying vessels in CAG images as normal or abnormal instead of providing a continuous percentage of severity for every stenosis^7,22^, and require human inputs to assist with the interpretation^2,3,14,21^. These limitations make them less representative of real-world clinical data. For example, some focus only on specific projection angles^3^ or the simpler structure of the right coronary artery (RCA), avoiding the more complex left coronary artery (LCA)^3,14,22^. A recent method, CathAI^5^, automates the assessment of stenosis severity from CAG images. However, its algorithm for identifying coronary artery segments has shown suboptimal performance, indicating a need for improvement in accurately assigning stenoses to the correct segment. Additionally, the methods mentioned previously rely on static images instead of dynamic videos, potentially missing critical information that clinicians frequently derive from video analysis to evaluate stenosis severity^5^.

In this work, as a primary objective, we aimed to develop a video-based algorithmic pipeline called DeepCoro, which goal is to automatically localize stenosis and assess their severity in CAG videos of both the LCA and RCA, and compare its performance to cardiologists’ visual assessments made on a large real-world CAG dataset spanning 5 years from the Montreal Heart Institute (MHI). DeepCoro is a cutting-edge pipeline that leverages videos instead of static images for the automatic evaluation of CAGs. Distinguished by its innovative coronary artery segment recognition and video-based stenosis percentage prediction algorithms, DeepCoro aims to enhance diagnostic accuracy by using the temporal dimensions present in CAG videos, mimicking the comprehensive assessment performed by cardiologists. Leveraging and enhancing the anatomic structure detection and stenosis detection algorithms from CathAI^16^, our suite incorporates substantial innovations: a registration algorithm that pioneers a new technique for tracking vessels and aligning videos—a key factor in CAG video analysis; a segmentation algorithm that stands out for its ability to segment CAG images, as evidenced by recent public dataset performances^23^, offering clear insights into the relationships among coronary artery segments; a coronary artery segment assignment algorithm that introduces a more accurate method for identifying artery segments compared to existing approaches; and a stenosis percentage algorithm, pioneering the application of video-based AI models for stenosis prediction and achieving higher accuracy that closely matches the diagnostic performance of cardiologists. Together, these algorithms signify a leap forward in the automated analysis of CAG videos. As secondary objectives, we aimed to benchmark the effectiveness of DeepCoro against an existing state-of-the-art image-based pipeline, CathAI^5^, which was re-trained on the same dataset. Additionally, we aimed to evaluate the performance of DeepCoro against existing CAG evaluation methods, concentrating on its consistency relative to human evaluators and its correlation with QCA labels.

**Fig. 1:**
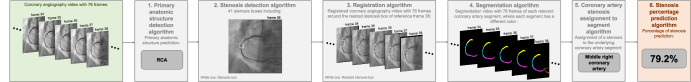
DeepCoro pipeline overview. Overview of DeepCoro’s algorithmic pipeline and example of outputs from each algorithm of a 76 frames coronary angiography video. In practice, steps 3 through 6 must be performed for all stenosis boxes detected at step 2, but the figure shows an example using frame 36 as a reference frame. White box: Stenosis localisation box. Green background box: DeepCoro’s input representing videos of left or right coronary angiograms. Grey background box: DeepCoro’s intermediary output. Orange background box: DeepCoro’s final output representing a continuous stenosis percentage as well as the underlying coronary artery segment with the stenosis. RCA Right Coronary Artery.

DeepCoro evaluates the severity of stenoses in CAG videos stored in Digital Imaging and Communications in Medicine (DICOM) format using a series of sequential algorithms. Its pipeline comprises six core algorithms (shown in Fig. 1), addressing various functionalities ranging from anatomical structure detection to stenosis percentage prediction; notably, four of these algorithms (Algorithms 3–6) are innovative additions introduced in this study. Algorithm 1 (the Primary Anatomic Structure Detection Algorithm) uses an Xception^24^ image-based model^5^ that distinguishes the primary anatomic structures, like the RCA, LCA, aorta, radial artery, left ventricle, catheter, and femoral artery, present in the most frames of the video. This algorithm was used to exclude videos not mostly containing the RCA or LCA from further analyses. Algorithm 2 (the Stenosis Detection Algorithm) uses the RetinaNet^25^ architecture, a state-of-the-art model for object detection, to pinpoint the location of coronary artery segments and stenoses. It achieves this by drawing bounding boxes around these areas, thereby defining their precise coordinates. Algorithm 3 (the Registration Algorithm) aims to align the stenosis bounding box derived by Algorithm 2, resized for consistency across videos, given the inherent motion of cardiac structures during systole or breathing. To achieve this, spatial translations were used for aligning the stenosis box in a reference frame to previous and subsequent frames, guided by a Discriminative Correlation Filter^26^. Algorithm 4 (the Segmentation Algorithm) segments full videos by applying an ensemble of seven segmentation models frame by frame, to generate registered multi-class segmented videos depicting 11 epicardial coronary artery segments. Algorithm 5 (the Coronary Artery Stenosis Assignment to Segment Algorithm) pinpointed the coronary artery segment affected by stenosis. It examined the central pixels of the resized stenosis-indicative region of the segmented registered video in each frame, and matched them with the coronary segments identified by Algorithm 4. Algorithm 6 (the Stenosis Percentage Prediction Algorithm) uses a modified version of the Swin3D^27^ architecture, a state-of-the-art video classification transformer model, adapted for regression tasks to output stenoses percentages ranging between 0 to 100%.

## Results

We introduce DeepCoro, a state-of-the-art pipeline for interpreting CAG videos, benchmarked against the clinical gold standard of visual assessment of stenoses by experts. Our main objective was to describe the accuracy of DeepCoro benchmarked to these human interpretations. Additionally, we compared DeepCoro’s capabilities with CathAI, setting both against the benchmark of expert human analysis to underline the efficacy and advancement of our approach over current state-of-the-art approach, CathAI.

### Performance of the DeepCoro pipeline

**Fig. 2:**
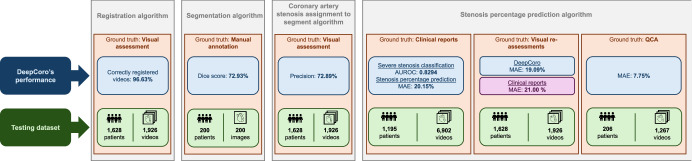
Main findings uncovered through DeepCoro’s four algorithms. DeepCoro’s performance and definition of the datasets and ground truth on which this performance was calculated for the fours algorithms introduced with DeepCoro (Algorithm 3–6). Blue box: Quantification of DeepCoro’s performance. Green box: Description of the testing dataset. Orange box: Identification of the ground truth used as reference to generate the performance metrics. Grey box: Identification of the algorithm being evaluated. Purple box: Quantification of the performance of clinical reports. AUROC Area Under the Receiver Operating Curve, MAE Mean Absolute Error.

To assess the efficacy of the DeepCoro pipeline, we evaluated the performance of its component algorithms (Algorithms 3–6) as well as our PCI removal algorithm. The main findings obtained with DeepCoro’s pipeline are summarized in Fig. 2. Dataset A was a central dataset for training and evaluating DeepCoro. We had 182,418 CAG videos in DICOM format of the LCA and RCA in our MHI clinical database which, after applying Algorithm 1–5, and after excluding videos of PCI and patients with previous CABG, resulted in 44,138 cropped videos of stenosed coronary artery segments that formed Dataset A (see Supplementary Fig. 6 for more details on the exclusions). Average age of patients was 67.6±11.0 years old, and 12,917 (29%) were identified as female and 30,067 (68%), as male. The average stenosis percentage was 20.6 ± 30.2% and we had 16% severe stenoses. The relatively low mean and wide standard deviation (SD) reflect a wide spectrum of stenosis percentages, including a substantial proportion of segments with 0% stenosis as identified by cardiologists, while our stenosis detection algorithm detected one.

A significant challenge in analyzing CAG videos is the movement of vessels within the videos, which can complicate the analysis of specific areas of interest. Algorithm 3 successfully registered 96.63% of the videos, as verified by expert annotators in Dataset B, a subset of our clinical database. The videos that were not correctly registered mostly either contained obstructive background elements, such as pacemaker leads or sternotomy wires, or suffered from poor contrast injections.

For the multi-vessel segmentation algorithm (Algorithm 4), our best performance was observed using an ensemble of seven models, averaging the predictions across the different models. We obtained a Dice score, PPV and sensitivity of respectively 73.93%, 75.96% and 70.12% using a weighted average across segments in the test set of a manually segmented dataset (Dataset C) (Supplementary Table 4). This suggests that there is a strong agreement and significant overlap between the predicted segmentations and ground truths. For coronary artery segment prediction, we obtained similar performances against human annotators on Dataset B for Algorithm 5 with a positive predictive value (PPV), sensitivity and F1-score across all 11 coronary segments of 71.89%, 70.72% and 70.71% respectively (Supplementary Table 5). Owing to the pipeline’s high accuracy for upstream algorithms, it was subsequently applied to our dataset for the training and inference stages of Algorithms 6.

We optimized DeepCoro’s PCI removal algorithm for high sensitivity to ensure accurate identification and exclusion of PCI-related videos, as PCIs can greatly increase error rates in the stenoses labelling, due to plaque modification. Our PCI removal algorithm is comprised of two thresholding methods and, using Youden’s index, we determined the optimal cut-offs to be 0.16 for Method 1 and a 25-minute offset for Method 2. Individually, Method 1 showed a sensitivity of 91.89%, and Method 2, 79.73% and, when combined, the sensitivity increased to 95.27%, indicating that the integrated approach effectively identified most video recordings of PCI procedures for subsequent removal. After determining that the registration, multi-vessel segmentation and PCI removal algorithm were working properly, we excluded all videos that were associated to PCI or post-PCI (*n* = 57,040 videos) and patients that underwent a CABG (*n* = 1895 videos) from the MHI clinical database.

Next, Algorithm 6 demonstrated high classification performance and moderate correlations for both vessels on Dataset A using the Swin3D approach compared to other video-classification models (Supplementary Table 11). Specifically, the RCA exhibited higher stenosis severity classification performance (AUROC = 0.8643; 95% CI: 0.8537–0.8745), sensitivity (76.20%; 95% CI: 73.98–78.60), and precision-recall balance (AUPRC = 0.5578: 95% CI: 0.5242–0.5890), along with a stronger correlation coefficient (*r* = 0.6200; 95% CI: 0.6018–0.6372) for stenosis percentage regression task (Table 1). These results suggest that the model is more adept at identifying and quantifying severe stenoses in RCA exams than LCA. On average, it requires 62.60 seconds for DeepCoro to complete an end-to-end analysis of a DICOM video on a RTX3090 GPU (Supplementary Table 14). The main findings obtained with DeepCoro’s pipeline are summarized in Fig. 2.

**Table 1:** Artery-level performance of DeepCoro in the test set of Dataset A

Task	Metric	Coronary artery		
		*LCA*	*RCA*	*LCA* + *RCA*
Number of exams		2568	2259	4827
Number of severe stenoses, ≥70% (*n* (%))		536 (21%)	345 (15%)	881 (18%)
Number of non-severe stenoses, <70% (*n* (%))		2032 (81%)	1914 (85%)	3946 (82%)
Number of healthy vessels, 0% stenoses (*n* (%))		1253 (49%)	1075 (48%)	2328 (48%)
Classification^a^	AUROC	0.8017 (0.7919–0.8124)	0.8643 (0.8537–0.8745)	0.8294 (0.8215–0.8373)
	AUPRC	0.5092 (0.4868–0.5329)	0.5578 (0.5242–0.5890)	0.5239 (0.5041–0.5421)
	Sensitivity (%)	70.70 (68.75–72.73)	76.20 (73.98–78.60)	72.86 (71.24–74.47)
	Specificity (%)	74.51 (73.56–75.43)	79.03 (78.10–80.04)	76.71 (76.05–77.36)
	PPV (%)	41.06 (39.48–42.70)	37.08 (35.11–39.00)	39.42 (38.15–40.68)
	F1-score (%)	51.95 (50.32–53.58)	49.88 (47.86–51.78)	51.15 (49.81–52.39)
Regression	MAE (%)	22.19 (21.82–22.52)	17.82 (17.48–18.16)	20.15 (19.88–20.40)
	*r*	0.4890 (0.4704–0.5087)	0.6200 (0.6018–0.6372)	0.5497 (0.5360–0.5630)

*AUPRC* Area Under the Precision-Recall Curve, *AUROC* Area Under the Receiver Operating Curve, *LCA* Left Coronary Artery, *MAE* Mean Absolute Error, *PPV* Positive Predictive Value, *r* Pearson’s correlation coefficient, *RCA* Right Coronary Artery.

^a^DeepCoro predictions were binarized with a threshold of 0.23, as determined on the validation set.

The performance at the artery-level of the DeepCoro’s pipeline on the test set of Dataset A. The range in parentheses is the 95% confidence interval generated by bootstrapping.

In Supplementary Figs. 3a and 4a, DeepCoro’s predictions are compared to the actual stenosis percentages for Dataset A, demonstrating a consistent linear correlation between predicted and true stenosis values. Supplementary Table 8 presents stratified results by age groups and sex, revealing no significant differences in performance based on sex. DeepCoro showed higher accuracy for single stenosis cases than for multiple stenoses (Supplementary Table 13). Specifically, for the RCA, the area under the receiver operating characteristic curve (AUROC) was 0.8988 (95% CI: 0.8839–0.9166) in single stenosis scenarios compared to 0.7999 (95% CI: 0.7878–0.8129) for multiple stenoses. Similarly, for the LCA, the AUROC was 0.8493 (95% CI: 0.8259–0.8754) for single stenosis cases versus 0.7542 (95% CI: 0.7447–0.7634) for multiple stenoses. Supplementary Table 15 highlights DeepCoro’s performance disparity in various scenarios: a mean absolute error (MAE) of 19.27% for diagnostic exams, increasing to 21.44% for videos identified as PCIs and to 23.86% for patients with previous CABG, demonstrating the rationale behind excluding these more complex and variable cases from our training set.

### Comparison with the CathAI pipeline

In a head-to-head comparison of CAG interpretation tools, DeepCoro significantly outperformed CathAI^5^. The evaluation focused on stenosis assignment to coronary artery segments and stenosis percentage prediction performances. DeepCoro’s segmentation method demonstrated a more accurate stenosis assignment, achieving a higher overall average PPV (71.89% versus 59.10%), sensitivity (70.72% versus 56.50%), and F1-score (70.71% versus 56.50%) than CathAI’s bounding box approach. These results were consistently robust across individual segments in Dataset B, indicating DeepCoro’s reliable performance enhancement without significant variance, as detailed in Supplementary Table 5.

Secondly, comparing stenosis classification in combined LCA and RCA, DeepCoro’s video-based approach demonstrated significant improvements over CathAI with AUROC values at the artery-level of 0.8294 (95% CI: 0.8215–0.8373) versus 0.7953 (95% CI: 0.7875–0.8038) (*p* < 0.01, as determined by DeLong’s test) and area under the precision-recall curve (AUPRC) values of 0.5239 (95% CI: 0.5041–0.5421) versus 0.4670 (95% CI: 0.4497–0.4849) (*p* < 0.01, as determined by DeLong’s test). In addition, Algorithm 6 (DeepCoro’s video-based model) had lower variability with the report stenosis, with a MAE of 20.15% (95% CI: 19.88–20.40) versus 21.61% (95% CI: 21.35–21.87), and higher correlation, with a *r* of 0.5497 (95% CI: 0.5360–0.5630) versus 0.4571 (95% CI: 0.4430–0.4711). We present a comparative performance table at the artery-level and video-level for both approaches in Supplementary Tables 6 and 7 respectively which demonstrates superior performance for DeepCoro’s video-based model over the CathAI’s image-based model.

### Inter-observer variability of visual assessment methods

In the expert-reannotated Dataset B, DeepCoro demonstrated an AUROC of 0.8699 (95% CI: 0.8564–0.8853) and an AUPRC of 0.7042 (95% CI: 0.6717–0.7339) for combined LCA and RCA in stenosis severity classification, surpassing the clinical reports which showed an AUROC of 0.7533 (95% CI: 0.7328–0.7744) and an AUPRC of 0.4737 (95% CI: 0.4382–0.5086) (Table 2). For regression tasks, DeepCoro’s MAE was 19.09% (95% CI: 18.55–19.58) with a *r* of 0.6792 (95% CI: 0.6598–0.7004), in contrast to clinical reports with an MAE of 21.00% (95% CI: 20.20–21.76) and a *r* of 0.5000 (95% CI: 0.4702–0.5302; Table 2; Supplementary Figs. 3b and 4b). Overall, DeepCoro’s performance was closer to two experts annotating the CAG videos, rather than the clinical report, demonstrating DeepCoro’s potential as a standardized approach to assess CAG videos. Ultimately, DeepCoro’s Algorithm 6 accuracy more closely mirrored the assessments of two expert cardiologists in annotating CAG videos and was superior to the conventional clinical report.

**Table 2:** Video-level performance of coronary angiography clinical reports and DeepCoro against expert annotated videos present in Dataset B

Task	Metric	Coronary artery					
		*LCA*		*RCA*		*RCA* + *LCA*	
		*Clinical reports*	*DeepCoro*	*Clinical reports*	*DeepCoro*	*Clinical reports*	*DeepCoro*
Number of videos		475		490		965	
Number of severe stenoses, ≥70% (*n* (%))		105 (22%)		92 (19%)		197 (20%)	
Number of non-severe stenoses, <70% (*n* (%))		370 (78%)		398 (81%)		768 (80%)	
Number of healthy vessels, 0% stenoses (*n* (%))		335 (71%)		322 (66%)		657 (68%)	
Classification	AUROC	0.7430 (0.7136–0.7735)	**0.8781 (0.8608–0.8965)**	0.7649 (0.7359–0.7959)	**0.8599 (0.8386–0.8867)**	0.7533 (0.7328–0.7744)	**0.8699 (0.8564–0.8853)**
	AUPRC	0.4908 (0.4400–0.5369)	**0.7066 (0.6659–0.7494)**	0.4684 (0.4168–0.5213)	**0.7040 (0.6592–0.7483)**	0.4737 (0.4382–0.5086)	**0.7042 (0.6717–0.7339)**
	Sensitivity (%)	46.63 (41.57–51.76)	**79.91 (76.54–83.95)**	47.88 (42.31–52.78)	**77.20 (72.86–81.94)**	47.30 (43.95–50.64)	**78.74 (76.05–81.70)**
	Specificity (%)	**91.87 (90.57–93.27)**	77.05 (75.00–79.12)	**93.73 (92.63–94.94)**	77.40 (75.39–79.50)	**92.81 (91.95–93.77)**	77.20 (75.78–78.60)
Regression	MAE (%)	22.28 (21.18–23.39)	**19.76 (19.02–20.53)**	19.79 (18.74–20.84)	**18.45 (17.75–19.15)**	21.00 (20.20–21.76)	**19.09 (18.55–19.58)**
	*r*	0.4661 (0.4221–0.5084)	**0.6653 (0.6406–0.6916)**	0.5332 (0.4929–0.5757)	**0.6914 (0.6624–0.7232)**	0.5000 (0.4702–0.5302)	**0.6792 (0.6598–0.7004)**

Video-level performance of DeepCoro and clinical reports on Dataset B. The statistically significant metrics where the confidence intervals don’t overlap are shown in bold. DeepCoro predictions were binarized with a threshold of 0.23, as determined on the validation set, and clinical used a threshold of 0.7 for sever stenosis classification. The range in parentheses is the 95% confidence interval generated by bootstrapping.

*AUPRC* Area Under the Precision-Recall Curve, *AUROC* Area Under the Receiver Operating Curve, *MAE* Mean Absolute Error, *r* Pearson’s correlation coefficient, *RCA* Right Coronary Artery, *LCA* Left Coronary Artery.

### Re-training and performance of DeepCoro on the MHI QCA dataset – dataset D

To verify if the performance of our model mirrors the lower variability of QCA, we fine-tuned and tested the regression performance of our pipeline on Dataset D, a dataset of CAG videos which uses QCA assessment as ground truth. The average percentage annotated in this dataset is 33.7 ± 11.7%. Overall, the fine-tuned model had a MAE against QCA of 7.75% (95% CI: 7.37–8.07; Supplementary Table 9; Supplementary Figs. 3c and 4c).

## Discussion

In the current study, we introduced DeepCoro, a ground-breaking video-based pipeline for the interpretation of CAG videos. Our approach describes an innovative ensemble segmentation approach, artery tracking algorithm and stenosis percentage prediction algorithm which mark a significant advancement in the automated analysis of CAG videos. We demonstrated good classification and regression performance for stenosis severity assessment on a large real-world dataset, spanning 5 years and over 40,000 CAG stenosis videos. Second, when benchmarked against the existing image-based CathAI pipeline^5^, DeepCoro demonstrated superior performance, setting a new standard for state-of-the-art automatic CAG interpretation. Third, DeepCoro’s performance not only aligned more closely with the expert evaluations from seasoned cardiologists but also exhibited lower variability when compared to clinical reports, enhancing the reliability of CAG video assessments. Fourth, our findings underscore DeepCoro’s versatility, showing its ability to be fine-tuned for diverse applications, such as QCA assessments, where it displayed acceptable classification accuracy. Finally, our model weights were made publicly available which will accelerate the research in this field by enabling researchers and cardiologists to fine-tune them to their own dataset and develop distinctive applications.

During the evaluation of DeepCoro’s Algorithm 6 for stenosis prediction, we noted a MAE of 20.15% (95% CI: 19.88–20.40), which falls within the typical variability range (6.9–26.5%^28^) noted among practitioners as reported in the literature. By aligning Algorithm 6 with the consensus annotations from two veteran interventional cardiologists, we reduced its variability to 19.09% (95% CI: 18.55–19.58), outperforming the inter-observer variability of 21.00% (95% CI: 20.20–21.76) found within this dataset. The precision of Algorithm 6 was further enhanced through calibration with QCA assessments. This fine-tuning highlights DeepCoro’s capability to diminish variability and improve the accuracy of stenosis evaluations, with results reflecting the nature of the training or fine-tuning dataset. DeepCoro’s benefits extend beyond its capacity to reduce variability. The algorithm’s design also allows for scalability across different datasets and adaptability to new diagnostic criteria, potentially setting a new standard for reproducibility in CAG interpretation. Its application could lead to more consistent and reliable stenosis assessments, by acting as an independent observer in the interpretation of CAG, which could lead to better-informed clinical decisions and potentially improving patient outcomes by ensuring a higher degree of diagnostic accuracy.

To maintain DeepCoro’s accuracy and reliability, excluding videos of PCI and excluding patients with previous CABG is critical. Our PCI exclusion algorithm, with a sensitivity of 95.27%, effectively filtered out most PCI interventions, a key step in enhancing model precision and training performance. DeepCoro’s efficacy is notably reduced in PCI and CABG cases, especially for RCA assessments (Supplementary Table 15). By utilizing these exclusion algorithms, we minimize biases and inaccuracies, thereby improving DeepCoro’s reliability for clinical application (Supplementary Table 11). This approach ensures DeepCoro remains a dependable tool for diagnosing coronary artery disease, free from the distortions caused by procedural modifications

DeepCoro demonstrated an AUROC of 0.8294 (95% CI: 0.8215–0.8373) on a comprehensive real-world dataset, aligning with prior stenosis classification research but significantly expanding on the scope with data spanning five years. This contrasts with earlier studies that used under 500 annotated angiogram frames, but obtained a higher AUC of 0.97^22^. Au et al. achieved an AUROC of 0.825 for their stenosis severity classification algorithm alone applied solely on RCA videos, but their performance declined when they applied their pipeline end-to-end to automatically interpret angiograms^14^. Notably, our end-to-end pipeline maintained a comparable performance for both RCA and LCA videos, matching the AUROC scores of algorithms tested solely on less complex RCA images^14^. Zhao et al. reported a sensitivity of 55.56% for severe stenosis classification, lower than our method’s 67.64% (95% CI: 66.09–69.31), highlighting our superior performance in identifying severe stenoses^21^. Our study’s strengths include a much larger patient cohort of 8057 compared to Zhao et al.’s 99, and the use of all 11 projection angles compared to Zhao et al. who used 5, significantly enhancing the clinical applicability and robustness of our findings^21^. Zhou et al. achieved a MAE of 15.9 ± 13.3% in a study focusing on RCA stenoses among 102 patients^3^. This highlights DeepCoro’s wider clinical applicability and robustness. Our model’s accuracy, combined with a diverse and extensive real-world dataset, offers superior generalizability compared to earlier studies that were restricted by smaller patient numbers and limited scope in terms of views analyzed.

Perhaps the most comprehensive work involved an algorithmic pipeline called “CathAI” that was trained on 13,843 studies spanning 5 years of data. They achieved an AUC of 0.862 (95% CI: 0.843–0.880)^5^. CathAI, while a state-of-the-art pipeline, had several limitations, notably its reliance on static images rather than video data. This approach may overlook crucial temporal information, which is essential for accurate diagnosis. Video-based models, by leveraging the inherent variability in multiple cardiac cycles, enhance diagnostic precision and effectively address the fluctuations in cardiac function that occur from one heartbeat to the next^29^. For instance, EchoNet-Dynamic, a video-based deep learning algorithm, exemplifies this advancement by outperforming human experts and image-based models in key diagnostic tasks like left ventricle segmentation and ejection fraction estimation from echocardiographic videos, showcasing the potential for video models to improve reproducibility and precision in cardiovascular disease diagnosis^29^. DeepCoro addressed these challenges by using a video-based analysis framework, effectively capturing the dynamic nature of cardiac cycles, and thereby enabling accurate stenosis estimation. Indeed, besides the technical advantages of video-based models, the inclusion of temporal aspects in video analysis offers significant benefits over still images, particularly in understanding complex phenomena like stenosis morphology and its effects on blood flow. Stenoses may exhibit varying behaviors at different phases of the cardiac cycle due to the dynamic nature of the cardiovascular system, which is important to consider during their analysis in a CAG video. Video analysis provides a more comprehensive and nuanced perspective of CAG videos, mirroring the way clinicians perceive and analyze angiograms, while still images-based algorithms lack sequential data analysis, which is crucial for studying dynamic physiological processes. In our study, DeepCoro consistently surpassed CathAI^5^ in regression metrics across all coronary arteries and matched or outperformed it in most classification metrics. The system proved especially effective in assessing the LCA, demonstrating the superior capability of video-models in analyzing complex anatomical structures. DeepCoro excelled in accurately assigning stenoses to coronary segments, significantly reducing data mislabeling. This contrasts with CathAI’s RetinaNet approach, which sometimes incorrectly interpreted the coronary artery tree’s structure by including irrelevant background details in its predictions. Unlike DeepCoro’s holistic analysis of the vessels, CathAI identifies vessel segments using bounding boxes but fails to connect artery segments, ignoring the vessel’s anatomy. This method assigns stenoses to bounding boxes without considering the interconnected nature of the coronary artery tree, potentially compromising accuracy (Supplementary Table 12). In contrast, DeepCoro’s segmentation method effectively recognized the interconnectedness of artery segments. This approach is more aligned with the methods cardiologists use, assessing artery segments based on their positions within the coronary artery tree, thereby enhancing stenosis prediction precision and the overall robustness of the algorithm.

Direct comparisons between DeepCoro and other models in existing literature present challenges, primarily due to the unique and extensive dataset that our approach used, a dataset not commonly used for testing other methods. We rigorously applied DeepCoro to a comprehensive collection of videos, covering both the RCA and LCA from all projection angles. This approach was undertaken without imposing extensive exclusion criteria, ensuring a broad and representative dataset that accurately reflects real-world clinical scenarios. In contrast, other models in the field often rely on partial automation or restrictive selection criteria, which may not capture the full spectrum of clinical scenarios, making DeepCoro a more comprehensive and clinically pertinent tool.

We also demonstrated that DeepCoro can be applied to new tasks. Upon fine-tuning with video annotations derived from QCA, DeepCoro achieved a reduced MAE of 7.75% (95% CI: 7.37–8.07%), aligning with the reduced variability typically associated with QCA compared to visual assessment. This MAE is notably less than the 10–17% variability range reported in literature when comparing QCA annotations with visual assessments^30^. Such approach could be undertaken in the future to fine-tune DeepCoro for calcium estimation, identifying the vulnerable plaque^31^ or predicting the physiological impact of stenoses^32^.

Results stratified across age groups and sexes demonstrate no significant bias on Dataset A’s test set (Supplementary Table 8), although the best performance was observed at extremes of age and those aged 60–75 had slightly reduced performance. Some differences in performance may be due to the varying proportion of severe stenoses across groups. Moreover, as individuals age, their vessels typically become stiffer due to increased calcification and changes in the vessel walls^33^. This age-related transformation in the coronary arteries’ structure can influence the diagnostic process and the accuracy of interpretations derived from CAG videos.

DeepCoro consistently performs better in cases with a single stenosis while maintaining moderate classification performance in handling videos featuring multiple significant stenoses, as detailed in Supplementary Table 13. This achievement sets DeepCoro apart as a cutting-edge tool to reliably excel across various complexities—where past automated methods struggled with accurate segment identification^16^ or relied solely on the analysis of images with a single diseased vessel^34^. Its adeptness at interpreting videos of various complexity, including multiple stenoses within the coronary artery tree signifies a substantial leap forward in the automated analysis of CAG videos. Moreover, we’re pioneering the initiative to openly share model weights, a step that promises to expedite progress in this research domain. By providing access to these weights, cardiologists may fine-tune the models to their specific datasets and foster the development of new applications for interpretation of CAGs.

As next steps, conducting a randomized controlled trial (RCT) to compare revascularization decisions based on AI-assisted CAG interpretations versus traditional methods will be key in understanding DeepCoro’s effect on clinical outcomes. For example, a RCT could be designed to assess the effectiveness of DeepCoro in detecting intermediate coronary stenoses (50–69%). This trial would require mandatory FFR testing for stenoses identified as intermediate by DeepCoro, examining its impact on key patient outcomes, such as all-cause mortality and myocardial infarction^19,35^. Patients undergoing coronary angiography could be randomly assigned to either the intervention group, receiving DeepCoro analysis followed by FFR-guided revascularization for identified stenoses, or the control group, subjected to standard care without DeepCoro’s assistance. This design could rigorously evaluate whether incorporating DeepCoro into clinical workflows can improve the precision of stenosis detection, thereby optimizing treatment decisions and potentially enhancing patient outcomes compared to conventional diagnostic approaches.

Understanding the limitations of DeepCoro is crucial for a comprehensive evaluation of its capabilities. A primary limitation is that the stenosis percentage used for training and testing is based on clinician interpretation from CAG videos, which may not always align with the actual stenosis values. This discrepancy underscores the potential benefit of employing large-scale datasets analyzed through objective measures like QCA to improve reproducibility. Despite this, our approach, when compared to the clinical report in Dataset B, had lower variability, suggesting that DeepCoro could be used to reduce variability in CAG interpretation. Of note, DeepCoro tends to underestimate stenosis severity reflecting the underlying stenosis distribution, likely due to the high prevalence of non-severe stenoses in the training dataset. Also, the current version of DeepCoro is optimized for detecting stenoses in 11 specific coronary segments, excluding side branches, and on patients that did not receive a CABG surgery. This decision to exclude side branches was influenced by the limited number of segmented instances available for these vessels, highlighting a notable limitation of our dataset and methodology and underscoring the need for future research to enhance the comprehensiveness of our approach^36^. Critically, only 5.4% of videos were identified as non-analysable and were excluded because they lacked identifiable coronary artery segments, according to Algorithm 5 (Supplementary Fig. 6). This demonstrates that a significant majority of our dataset’s videos are analysable, underscoring DeepCoro’s extensive capability for coronary angiography video evaluation. The current processing time for DeepCoro, averaging 62.60 s per DICOM image to generate predictions, is manageable but could be a hurdle in clinical settings where quick turnaround is essential. To improve time efficiency, we are investigating methods to speed up processing, including enlarging batch sizes and implementing parallel computing techniques. These improvements are mainly technical challenges; overcoming them will enable DeepCoro’s smooth integration into clinical operations, thereby reducing potential delays. Furthermore, the pipeline’s reliance on multiple algorithms, each introduced a compounding degree of error, potentially affecting the final output’s quality. For example, our registration algorithm did not perfectly align all videos, with only 96.63% correctly registered, suggesting a need for further refinement to achieve consistent success across all cases. However, our approach remains the most comprehensive one, it underscores the different steps that must be taken to automatically interpret CAGs and these performances can be improved by annotating more data.

In conclusion, DeepCoro marks a substantial leap in CAG video interpretation. This multi-step video analysis pipeline adeptly mirrors the dynamic analysis conducted by cardiologists, offering a more standardized approach to CAG evaluation. It promises to standardize CAG assessments, potentially reducing clinical interpretation variability and is versatile enough for fine-tuning to new tasks such as the automated QCA measurement of angiograms. Future enhancements could enable DeepCoro to detect stenoses in complex coronary segments and evaluate critical features like calcification severity, or automated SYNTAX score, which is crucial for treatment planning. The next critical phase is to examine its effect on clinical decisions and assess its potential to improve patient care, particularly in revascularization strategies, possibly through a proposed RCT.

## Methods

DeepCoro employs a unique multi-step pipeline to detect and analyze stenosis in CAG videos stored in DICOM format. It leverages six specialized algorithms, where the data flows from one algorithm to the other. It has been trained and validated on an extensive dataset from MHI. DeepCoro’s architecture builds on the foundational work of CathAI^5^, integrating its essential algorithms for detecting primary anatomical structures and identifying stenosis (Algorithms 1 and 2; Fig. 1). These algorithms were adopted without further training on our dataset. Our original contribution is detailed in Algorithms 3–6, representing our advancements in this field^5^. DeepCoro initiates with the detection of primary anatomical structures (Algorithm 1), selectively focusing on videos pertaining to the RCA and LCA. It then employs RetinaNet^25^ models (Algorithm 2) to locate stenoses within these coronary segments. A registration algorithm (Algorithm 3) follows, aligning frames to account for heart and respiratory movement, creating a stable video in reference to a stenotic coronary segment. Our sophisticated multi-class segmentation algorithm (Algorithm 4) plays a pivotal role in the process by categorizing the coronary artery into proximal, middle, and distal segments. Algorithm 5, in each frame, evaluates the content of the resized stenosis box, focusing on pixels within the central region of the reference area, which are matched to the underlying coronary artery segment as predicted by Algorithm 4. This method is designed around the typical central placement of stenosis within annotations, thus concentrating on this area with the assumption that it houses the relevant segment. Lastly, a stenosis severity prediction algorithm (Algorithm 6), using a modified Swin3D^27^ transformer model, quantifies stenosis severity from the aligned video to predict a continuous percentage in targeted artery segments. This integrated approach facilitates automatic interpretation of CAG videos without any human input.

### Algorithm 1: Primary anatomic structure detection algorithm

During CAG, the operator can record videos which often encompass anatomical structures beyond the RCAs and LCAs, such as the aorta, radial artery, left ventricle, catheter, or femoral artery. Previously, as part of CathAI’s pipeline^5^, an Xception image-based model^24^ was trained and tested on 14,366 CAG images acquired at the University of California, San Francisco (UCSF) and separated by cardiologists in 11 classes representing primary anatomic structures (listed in Supplementary Table 3)^5^. 9887 (70%) of those images were used for training, 1504 (10%) were used for validation and 2975 (20%) were used for testing^5^. A video-level prediction was calculated by determining the most frequent frame-level prediction in a video^5^. This algorithm allowed to detect LCAs and RCAs with PPVs of 97% and 93% respectively on their test set^5^. The trained model was available for us to apply on our dataset. After applying this algorithm to our data, videos not identified as RCAs or LCAs at the video-level prediction were excluded from further analysis in the pipeline, ensuring focus on the relevant coronary structures.

### Algorithm 2: Stenosis detection algorithm

RetinaNet^25^ is one-stage object detection model that used a focal loss function to address class imbalance during training and detected the position of different objects in images by outlining them with bounding boxes and classified the boxes as a certain object. It has shown a state-of-the art performance for a multitude of object localisation task^25^. Previously, as part of CathAI’s pipeline^5^, RetinaNet models were trained and tested on UCSF data to detect 19 structures (listed in Supplementary Table 3), some of which are of interest in this study, i.e. our 11 coronary artery segments of interest, stenoses and procedural instruments associated to a PCI (i.e. guidewires, balloon and stents).

In the CathAI study^5^, three distinct RetinaNet^25^ models were developed, each trained on various combinations of RCA, LCA, and projection angles (extracted from the DICOM metadata). One RetinaNet model focused on the right anterior oblique cranial projection of the LCA, another concentrated on the left anterior oblique straight projection of the RCA, and a third covered all other projection angles for both the LCA and RCA. The training of these RetinaNet models adhered to the original parameter specifications and was done on a dataset comprising 2788 CAG images of the LCA and RCA from UCSF. These images were meticulously annotated by a board-certified cardiologist, marking the locations of 19 different structures. The dataset allocation involved using 90% for training and 10% for testing. Any predictions with a probability below 0.5 were discarded to maintain accuracy.^5^ These trained algorithms were made available for direct application to our dataset.

The RetinaNet models demonstrated a high proficiency in localizing stenoses, correctly identifying 93.3% of them in the UCSF test set. Detailed results showcasing the performance of these networks trained on CAG images were published in the original paper^16^. Supplementary Table 12 shows an example of the raw output of a RetinaNet^25^ model on our data. The RetinaNet models trained on CathAI’s dataset were deployed to analyze every frame of each video in our dataset, which includes videos of both the LCA and RCA. When a video was classified as pertaining to the RCA, as determined by Algorithm 1, the RetinaNet models were programmed to exclude predictions for LCA segments, and the inverse is true for LCA-identified videos.^5^ The RetinaNet models were applied to all frames of our LCA and RCA videos, as identified by Algorithm 1.

To streamline this process, specific elements were established to facilitate efficient and accurate processing:

- Reference frame: Frame within a video where there is a stenosis box identified.
- Reference area: Whole content of a stenosis box when it intersects with a coronary artery segment box in a reference frame with an intersection over union over 0.5 (preliminary coronary artery prediction).
- Segment of interest: Coronary artery segment part contained in a stenosis box.

Each detected reference area was preliminarily assigned to the respective artery segment it overlaps in a specific frame (Supplementary Table 12), which is the method used by CathAI^5^ to assign a coronary artery segment to a stenosis. To manage the dataset size and computational demands, particularly for Algorithm 3, we limited the dataset to include the central reference area per artery segment for each video. Typically, this was the frame closest to the video’s midpoint, where the dye’s intensity peaks. This approach was necessary to avoid an excessively large dataset with multiple repetitive instances of the same coronary artery segment stenosis, which would have significantly increased computational requirements.

### Algorithm 3: Registration algorithm

The motion of the myocardium during systole and diastole, coupled with the effects of breathing, can result in considerable movement of the vessels throughout a CAG video^7^. Because of this, the segment of interest within a stenosis box may not align consistently with the reference frame coordinates in different frames of the CAG video. Therefore, it is essential to maintain alignment of the segment of interest across all frames within each stenosis box video. To achieve this, each video undergoes a registration process, aligning it with the reference stenosis boxes using spatial translations within the video’s spatial dimension.

Before registration, every stenosis box is resized as a square with sides closest to 17.5 mm, using the real-life dimensions associated to the pixel spacing of every video, for standardization across examinations. The Discriminative Correlation Filter from the OpenCV Python library was used to perform registration^26^. This approach performed better than similar registration methods and comparatively to approaches making use of AI for registration while being much faster^37^. It tracks the spatial displacement of a segment of interest at a certain frame within a video in reference to the reference stenosis box and the frame was moved considering this displacement^37^. After displacement predictions were made, a one-dimensional uniform smoothing filter was applied to the prediction to avoid errors in predictions that lead to sudden major shift from frame to frame in a registration. Supplementary Fig. 1 shows a registration example from a frame within a video. A registered video was generated for each stenosis box obtained after Algorithm 2.

### Algorithm 4: Segmentation algorithm

The segmentation algorithm takes an image as input and full videos can be segmented by applying the algorithm to each image contained in it. In the DeepCoro pipeline, registered CAG videos were segmented to obtain registered multi-class segmentation maps of 11 coronary artery segments (5 for the RCA – i.e. proximal RCA, middle RCA, distal RCA, posterolateral branch from the RCA and posterior descending artery – and 6 for the LCA – i.e. left main artery, proximal left anterior descending artery (LAD), middle LAD, distal LAD, proximal left circumflex artery (LCX) and distal LCX). These artery segment stem from 25 coronary artery segments labels available with the ARCADE dataset which are based on SYNTAX score definitions^38^ and listed in Supplementary Table 3. We chose 11 specific coronary segments for analysis in DeepCoro, mirroring those identified by CathAI, to enable a direct comparison between the two systems, as part of our secondary objective. This selection process also involved excluding side branches such as obtuse marginal and diagonals that appear infrequently and suffer from limited visibility within the dataset^36^. Such exclusions help avoid inaccuracies in segmentation and simplify the analysis of the LCA, as these rarely segmented branches can hinder the generalizability and performance of the segmentation model model.^36^ This algorithm was developed by testing, over 200 epochs, nine state-of-the-art segmentation models, i.e. UNet^39^, UNet++^40^, MANet^41^, LinkNet^42^, FPN^43^, PSPNet^44^, DeepLabV3^45^, DeepLabV3+^46^ and PAN^47^, and five loss functions, i.e. Jaccard loss, Dice loss, focal loss, Lovasz loss and Tversky loss. Hyperparameter optimisation was done with random search over the batch sizes of 4, 8, 16, 32 and 64, and learning rates ranging from 1e−2 to 1e−5 with 5-fold cross validation using the same folds for every set of hyperparameters. Segmentation maps were generated by associating each pixel to the class with the highest probability in the prediction map outputted by the models. The ensemble of seven models resulting in the best Dice coefficient on the validation set were selected (Supplementary Table 10). The prediction from our final segmentation model consists in the average of the prediction maps from all seven models and then generating the segmentation map.

### Algorithm 5: Coronary artery segment recognition algorithm

This algorithm generates predictions based on the content of the resized stenosis box in each frame of the registered segmented CAG video. In every video frame, a prediction for the artery segment is made by identifying pixels within the reference area that align with the central region and correspond to the appropriate coronary artery (LCA or RCA), as determined by the primary anatomic structure identified in Algorithm 1 (Supplementary Table 12). This approach is rooted in the way stenosis box annotations are typically applied with the stenosis positioned centrally. Consequently, the algorithm is designed to focus on this central area, under the assumption that the stenosis is centrally located, and thus the segment of interest, will be located there.

If multiple artery segment pixels were equally distant to the center of the stenosis box, the predicted artery segment was the one that has the greatest proportion of these pixels. In rare cases where two or more artery segments were equally distant to the center and all have the most pixels equally distant to the center, the predicted artery segment was the one that had the lowest SYNTAX index^48^. If no segmentation pixels were contained within the stenosis box, no artery segment prediction was made. A prediction was made for the whole video with a majority vote among the image-level predictions within the video. When the algorithm is unable to predict any artery segments in a video, no further predictions are made in that video.

For assessing stenosis severity at the video-level, a targeted approach is employed: when multiple videos from the same DICOM file are related to an identical artery segment, only the central instance is retained for the final calculation of stenosis percentage. The remaining videos, which are effectively duplicates for the same coronary segment, are removed. While most of these duplicates are initially filtered out by Algorithm 2, there are instances where Algorithm 5 might reassign videos to the same segment. In such cases, only one video per segment is kept. This strategy ensures a thorough and accurate evaluation of stenosis severity.

### Algorithm 6: Stenosis percentage prediction algorithm

A modified architecture of the video-based model called Swin3D^27^ was trained and validates to predict the severity of a stenosis. We conducted extensive experimentation with various deep learning architectures – such as X3D, MViT and R(2 + 1)D – and dataset configurations to identify the most suitable model for our task, fine-tuning significant parameters. Further details on the selection process and a comparison with other architectures can be found in Supplementary Table 11. Due to the extensive size and complexity of our dataset combined with the time-consuming nature of video models training, we focused on architectures renowned for their effectiveness in video classification tasks, rather than exhaustively exploring all possible models and parameters. After careful evaluation, we selected the Swin3D model, which has demonstrated state-of-the-art performance across a range of tasks and is particularly well-suited for large-scale video classification tasks like ours^27^. Swin3D is a state-of-the art three-dimensional video classification transformer that has demonstrated state-of-the-art performance for various tasks^27^. Transformers have demonstrated great potential for stenosis assessment in comparison to convolutional networks, and work best using large datasets, which we have access to ref. ^2^.

Swin3D’s classification layer^27^ was modified to perform regression by changing the activation function to a Sigmoid function and changing the output size to get one continuous value between 0 and 1, corresponding to a stenosis percentage (Supplementary Video 1 shows an example of DeepCoro’s output). One hot encoded vector identifying the coronary artery to which the stenosis belong, and a normalized value of the patient’s age were concatenated to the last feature vector of the model. Swin3D^27^ was pretrained on the public datasets Kinetics400^49^ and ImageNetV1^50^ to allow faster convergence of the model. Then, it was trained on our training set consisting of registered CAG videos cropped around stenosis boxes for 50 epochs with a learning rate of 0.001, the stochastic gradient descent optimizer and the mean square error loss function. 24 frames centered around the reference area of each video were used during training and testing. Hyperparameter tuning was performed by finding the parameters that lead to the epoch with lowest loss on the validation set. The final model used to make the predictions was the one associated with the epoch having the lowest loss. For Swin3D^27^, hyperparameter tuning was performed with learning rates of 0.00001, 0.0001 and 0.001. Batch sizes were chosen to maximize memory usage on a 48 Gb GPU; a batch size of 4 was used for Swin3D^27^.

We performed a comparative analysis between the image-based CathAI method and our video-based DeepCoro strategy for stenosis assessment using the Dataset A to enable a direct comparison of both approaches. CathAI used an image-based Xception^24^ model to estimate stenosis severity predictions^5^. In order to properly compare the percentage of obstruction prediction algorithm from CathAI and DeepCoro without external influences, we replace CathAI’s coronary artery segment prediction algorithm with DeepCoro’s. For CathAI, stenoses identified by its algorithm were enclosed in bounding boxes, enlarged slightly, and resized to standardized dimensions for processing. Average predictions across individual frames where a stenosis bounding box-coronary segment pair was present provided video-level performance metrics to be compared against DeepCoro’s Algorithm 6. CathAI underwent a training regimen of 50 epochs, using a learning rate of 0.001, the RAdam optimizer^51^, a batch size of 12, and the mean square error as the loss function. Hyperparameter tuning was performed the same way as for DeepCoro.

### Description of datasets

**Fig. 3:**
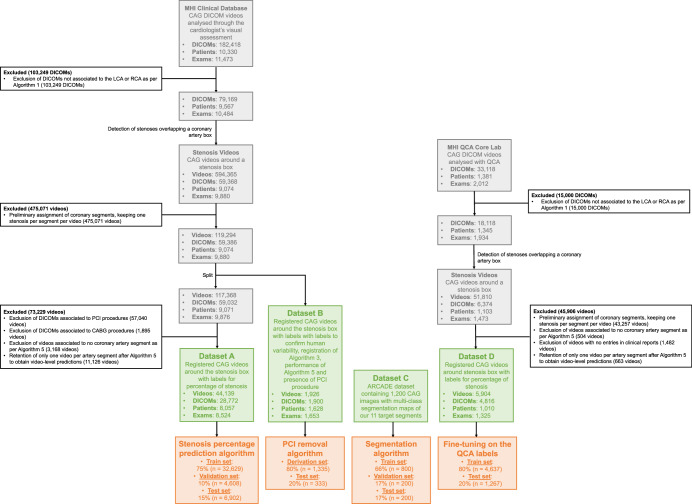
Datasets and patients used to train and validate DeepCoro. Datasets change in size when our algorithms are applied to our datasets. ARCADE is a public dataset described in ref. ^23^. White box: Exclusion details. Grey box: Intermediate datasets. Green box: Final datasets. Orange box: Dataset split for the development of an algorithm. ARCADE Automatic Region-based Coronary Artery Disease diagnostics using X-ray angiography imagEs, CABG Coronary Artery Bypass Grafting, CAG Coronary Angiography, DICOM Digital Imaging and Communications in Medicine, MHI Montreal Heart Institute, PCI Percutaneous Coronary Intervention, QCA Quantitative Coronary Angiography.

Our algorithms were trained and validated using four distinct datasets, detailed below, and illustrated in Fig. 3 (Supplementary Fig. 5 shows a detailed form of this figure). Comprehensive dataset characteristics and patient demographics are available in Supplementary Tables 1 and 2. All DICOMs were acquired at 15 FPS and a resolution of 512 pixels X 512 pixels on Philips, Siemens, and Toshiba X-ray machines with a low to moderate radiation intensity. We eliminated videos that did not feature the LCA or RCA, as identified by Algorithm 1. We further reduced the dataset due to computational constraints as outlined in the methodology associated with Algorithm 2, keeping only one video per DICOM per coronary artery segment preliminary predictions. Videos depicting PCI and CABG procedures were also removed. Any videos that did not display recognizable coronary artery segments as determined by Algorithm 5 were excluded. To facilitate video-level predictions, we maintained a single video for each artery segment in a DICOM after the application of Algorithm 5. All datasets comprise examinations from patients exhibiting a range of conditions: multiple stenoses, a single stenosis, or no stenosis at all. Detailed video counts for each scenario are provided in Supplementary Tables 1 and 2.

Dataset A was derived from the MHI’s comprehensive CAG videos database, which contains 182,418 videos of LCA or RCA in DICOM format, captured at 15 frames per second, and includes patients aged 18 years and older, spanning the period from January 1, 2017, to December 31, 2021. The dataset was used for the training, validation, and testing of DeepCoro. Clinical reports detail the percentage of coronary artery segment stenosis, ranging from 0 to 100%, associated to these videos as described by the interventional cardiologist after visual assessment. Segments with ≥70% stenosis were classified as severe, while those with less were labeled as non-severe. After applying Algorithms 1–5 to this database, we obtained a final dataset of 44,139 videos from 8057 patients with associated stenosis percentages from clinical reports. This dataset was partitioned into mutually exclusive training (75%), validation (10%), and test (15%) sets, ensuring that each patient could only belong to one of these subsets, for the training and evaluation of DeepCoro’s Algorithm 6.

Dataset B consists of 1926 videos of the LCA and RCA from 1628 patients, randomly selected from the MHI database. This selection occurred after the initial application of Algorithms 1–3. The purpose of this specific curation was to ensure that the dataset included only LCAs and RCAs with stenoses, facilitating the testing of our pipeline’s performance against a human-annotated dataset. All videos were simultaneously annotated by two cardiologists (interface shown in Supplementary Fig. 2) with over 10 years of experience in reading CAG videos to describe stenosis percentage (1926 labels), correctness of the artery segment registration (Algorithm 3; 1926 labels), identification of the coronary artery segment with a stenosis (1926 labels) and presence of PCI in the video (i.e. presence of a guidewire, balloon, or stent; 1668 labels). The purposes of this dataset were to assess inter-observer variability, determine the performance of Algorithm 3, 4 and 6, and develop an automatic algorithm to remove videos associated to PCI from our dataset. For the latter, we randomly divided the dataset into an 80% derivation set (to identify the optimal threshold for excluding PCI procedures) and a 20% hold-out test set where we tested this threshold to exclude PCI procedures. A video was deemed correctly registered when the targeted coronary segment remained in the predefined stenosis box from the onset of iodine dye visibility to its disappearance. For evaluating Algorithm 4’s accuracy, we excluded videos that did not correspond to the 11 pre-specified coronary segments as determined by both the algorithm and a cardiologist, removing 119 videos, and reducing the dataset from 1926 to 1807 videos. To assess the performance of Algorithm 4, the Dataset B was cleaned from PCI and CABG procedures with our algorithm; in total, 965 videos remained in Dataset B.

Dataset C is sourced from the Automatic Region-based Coronary Artery Disease diagnostics using X-ray angiography imagEs (ARCADE)^23^ public dataset and includes 1200 X-ray CAG images, with 25 different multi-class coronary artery segmentations. We only considered 11 epicardial coronary artery segments pertinent to our approach. Segments with very few annotated examples (i.e. diagonals or left posterior descending artery) were excluded. We randomly split 800 images (66% of the dataset) for Algorithm 4 training and 200 images each (17% of the dataset each) for validation and testing.

Dataset D was extracted from the MHI QCA Core Laboratory’s, which is a separate dataset from the MHI clinical database that features CAG videos from randomized controlled trials focused on lipid-lowering therapies^52^. This dataset comprised a unique patient population distinct from our primary clinical dataset, as it mainly contained mild-to-moderate coronary stenoses, with an average QCA stenosis severity of 33.7% ± 11.7%. These angiograms were recorded at 15 frames per second. Each angiogram underwent QCA analysis by trained technicians and was supervised by an expert physician. For this dataset, stenosis was categorized as severe with QCA stenosis percentage of ≥50%^30,53^. After applying Algorithms 1, 2, 3, and 5, the dataset was narrowed down from 33,118 CAG video to 5904 videos cropped around stenoses. This dataset provided an ideal setting to evaluate DeepCoro adaptability to different clinical contexts, using QCA stenosis percentage labels for retraining. Patients’ videos were randomly broken down into an 80–20% split, resulting into a training set of 4637 videos and a test set of 1267 videos for the external validation of DeepCoro.

### Removal of PCI procedures

The study aimed to ensure accurate labeling of CAG videos for diagnostic evaluation, prior to any plaque modification during a PCI. We excluded videos taken during or following a PCI because the stenosis observed could be modified during the intervention. This modification could lead to discrepancies between the video and the initial diagnostic stenosis labels. For example, a significant stenosis visualized during balloon angioplasty type PCI might appear less severe in the imaging captured during the procedure. Such intra-procedural changes in stenosis are usually not recorded; only the diagnostic stenosis percentage before PCI and the result after PCI are typically reported. However, we kept the initial diagnostic CAG videos of patients who subsequently underwent PCI treatments. To distinguish diagnostic CAG videos from those recording PCI, we developed a dual-method approach for identifying videos linked to PCI procedures:

1. Method 1: We used Algorithm 2 to identify frames containing procedural instruments indicative of a PCI (i.e. stent, balloon, guidewire). A metric *m1* was calculated by summing the number of frames containing the instruments according to RetinaNet^25^ predictions, then dividing by the total frame count. For a video, if *m1* was greater or equal to a pre-determined threshold, the video was identified as a PCI-related. This threshold was optimized using Youden’s index, applied to 100 equally spaced thresholds between 0 and 1 in a derivation set and then compared to the human annotations.
2. Method 2: We also leveraged clinical reports that specify the start and end times of each PCI procedure for both RCA and LCA views. Videos within these timeframes were flagged as PCI-related. Due to possible time discrepancies between clinical report timestamps and the video recording, an offset value *m2* was subtracted from each stenting event’s start time to account for inconsistencies. The optimal offset was determined by maximizing Youden’s index a derivation set, considering offsets of 0, 5, 10, 15, 20, 25, and 30 min.

The optimal thresholds for both Method 1 and Method 2 were initially determined using the derivation set of Dataset B. These thresholds were then validated on a separate hold-out test set of Dataset B. The combined approach associates a video to a PCI if one is detected with either of the two methods, for subsequent removal. By combining both methods, we systematically identified and excluded videos associated with PCI procedures, as well as any subsequent videos of a patient, from Dataset A. This ensures that DeepCoro is only trained on diagnostic CAG videos to make sure the labeling of coronary artery stenoses is the most accurate.

### Removal of patients with CABG

We also excluded videos connected to CABG procedures from Dataset A. This is due to the significant anatomical differences in patients who have undergone CABG surgery, which interferes with our objective of creating a universally applicable analysis pipeline for native coronary vessels. Clinical reports indicate whether a patient has undergone a CABG operation. Therefore, patients featuring bypassed vessels were excluded.

### Primary objective: assessment of the DeepCoro algorithm suite

Algorithm 1 and 2 performances were previously published^5^. Our current objective was to evaluate the original Algorithm 3–6 within the DeepCoro pipeline. For Algorithm 3, video registration performance was defined by the proportion of correctly registered videos, determined using human annotated data from Dataset B. Algorithm 4’s segmentation quality was validated on Dataset C’s test set by employing the Dice Score, PPV, and sensitivity for the delineation of the 11 targeted coronary artery segments. We also described sensitivity, PPV, and the F1-score for each of the 11 coronary artery segments of interest against ground truth labels from Dataset B. Algorithm 6’s efficacy in stenosis percentage prediction was gauged using MAE and Pearson’s correlation coefficient (*r*) on Dataset A’s test set. Its capability to classify stenosis severity (non-severe versus severe) was assessed using the AUROC, sensitivity, specificity, PPV, and the AUPRC. Clinician-reported stenosis percentages, which were dichotomized for severity classification at a 70% threshold, served as the gold standard for Dataset A, whereas a 50% threshold^30,53^ was used in Dataset D (evaluated through QCA). Due to important class imbalance favoring the non-severe stenosis class^54^, AUPRC and AUROC best represent DeepCoro’s performance for a comprehensive performance overview. Binarization of predictions used the optimal threshold derived from maximizing Youden’s index among 100 equally spaced thresholds between 0 and 1 on the validation set. Our primary performance was assessed at the artery-level which was derived by computing the mean stenosis severity for each coronary segment, using videos from the same patient taken on a single day. This approach provided an aggregated measure of stenosis for each artery segment, leveraging multiple angiographic views to enhance the robustness of the assessment.

We calculated 95% confidence intervals (CIs) using the bootstrapping method, which involves randomly selecting 80% of the test set data to recompute the performance metrics. This process was repeated across 1000 iterations, and the CIs were established from the resulting distributions of these metrics.

### Comparison with the CathAI pipeline

CathAI represents a notable advancement in AI-driven automated interpretation of CAGs^5^. However, it has limitations, such as suboptimal coronary artery segment prediction and reliance on still images, contrasting with the dynamic video analysis typically employed by cardiologists. Our goal was to compare our video-based DeepCoro pipeline with CathAI’s image-based one to assess if it could address these issues and enhance overall performance.

We first compared coronary artery segment predictions between CathAI and DeepCoro on Dataset B, using human annotations as the standard. CathAI’s approach, using RetinaNet^25^ models for stenosis identification, was evaluated against DeepCoro’s segmentation-based method (Algorithm 5). Performance metrics PPV, F1-Score, and sensitivity were computed for both systems.

Further, we analyzed stenosis severity on Dataset A, contrasting CathAI’s image-based model with DeepCoro’s video-based approach. To ensure a fair comparison for this task, we integrated DeepCoro’s coronary artery segment predictions into CathAI’s framework rather than using the coronary segment prediction from RetinaNet. CathAI’s stenoses, delineated with bounding boxes, were processed, and compared with DeepCoro’s Algorithm 6. Training parameters for CathAI, including epochs, learning rate, optimizer, and batch size, were aligned with DeepCoro’s settings. The comparison was based on uniform metrics, with model superiority determined by non-overlapping 95% CIs. We applied the DeLong test^55^ to evaluate statistical differences of AUROC and AUPRC between CathAI and DeepCoro for stenosis severity categorization, with a *p*-value below 0.05 indicating a significant difference.

### Assessment of inter-observer variability

To evaluate inter-observer variability, we conducted a comparative analysis between the performance of DeepCoro’s Algorithm 6 and the clinical reports annotations, against the average annotations from two expert interventional cardiologists with over ten years of experience provided in Dataset B. These annotations served as the ground truth, against which we compared the performance of Algorithm 6 as well as the accuracy of clinical reports. Clinical report predictions were obtained by retrieving the percentage of obstructions of the DICOM in clinical reports associated to the artery segment identified by the cardiologist in Dataset B, and were binarized with a 70% threshold. Key metrics such as the AUROC, AUPRC, sensitivity, specificity, MAE, and *r* were computed to quantitatively benchmark the performance of Algorithm 6 and clinical reports against the human annotated stenosis percentage.

### External validation against QCA

Fine-tuning is pivotal for adapting DeepCoro’s Algorithm 6 to new tasks, such as predicting stenosis percentages that match the inherently more consistent measurements from QCA. To test its capability for this precision-demanding task, we fine-tuned the algorithm using the train set of Dataset D—CAG videos annotated with QCA by the MHI core lab as the gold standard. The fine-tuning involved keeping the model’s parameters fixed except the last two linear layers which are adjusted over 100 epochs. We varied learning rates from 1e−2 to 1e−7 and determined the optimal rate to be 1e−3, based on the lowest loss achieved. The model, optimized for QCA annotations, was then rigorously evaluated on the test set to confirm its enhanced regression accuracy.

### Human subjects research

This study was reviewed and approved by the MHI Institutional Review Board. The need for individual informed consent was waived as it was deemed impractical to obtain consent from all patients present in this large dataset.

### Supplementary information

Supplemental Material

Supplementary Video 1

## Data Availability

The datasets analysed during the current study are not publicly available due to patient privacy concerns but may be available from the corresponding author on reasonable request, as feasible and permitted by the Montreal Heart Institute institutional review board. Algorithm 4 and 6 weights from our trainings can be accessible via this link https://huggingface.co/heartwise/DeepCoro.

## Code availability

The underlying code for DeepCoro’s inference of Algorithm 3–6 for this study is available in a GitHub repository and can be accessible via this link https://github.com/HeartWise-AI/DeepCoro.
