# Supplementary material for: Evaluation of stenoses using AI video models applied to coronary angiography
Source: NPJ Digit Med. 2024 May 23;7:138. doi: 10.1038/s41746-024-01134-4 (PMC11116436; doi:10.1038/s41746-024-01134-4)
Supplement: Supplementary file 1 — Supplemental Material [file 41746_2024_1134_MOESM1_ESM.docx]

# Supplementary Figures

**Supplementary Figure 1.** Example of a (a) reference frame, (b) unregistered frame N and (c) registered frame N within a video (white square represents the limits of the reference area)

**Legend**. Example of an unregistered frame within a video and its registered form in reference to the reference area. **White box**: Resized stenosis box.

**Supplementary Figure 2**. Interface for Dataset B Annotations.

**
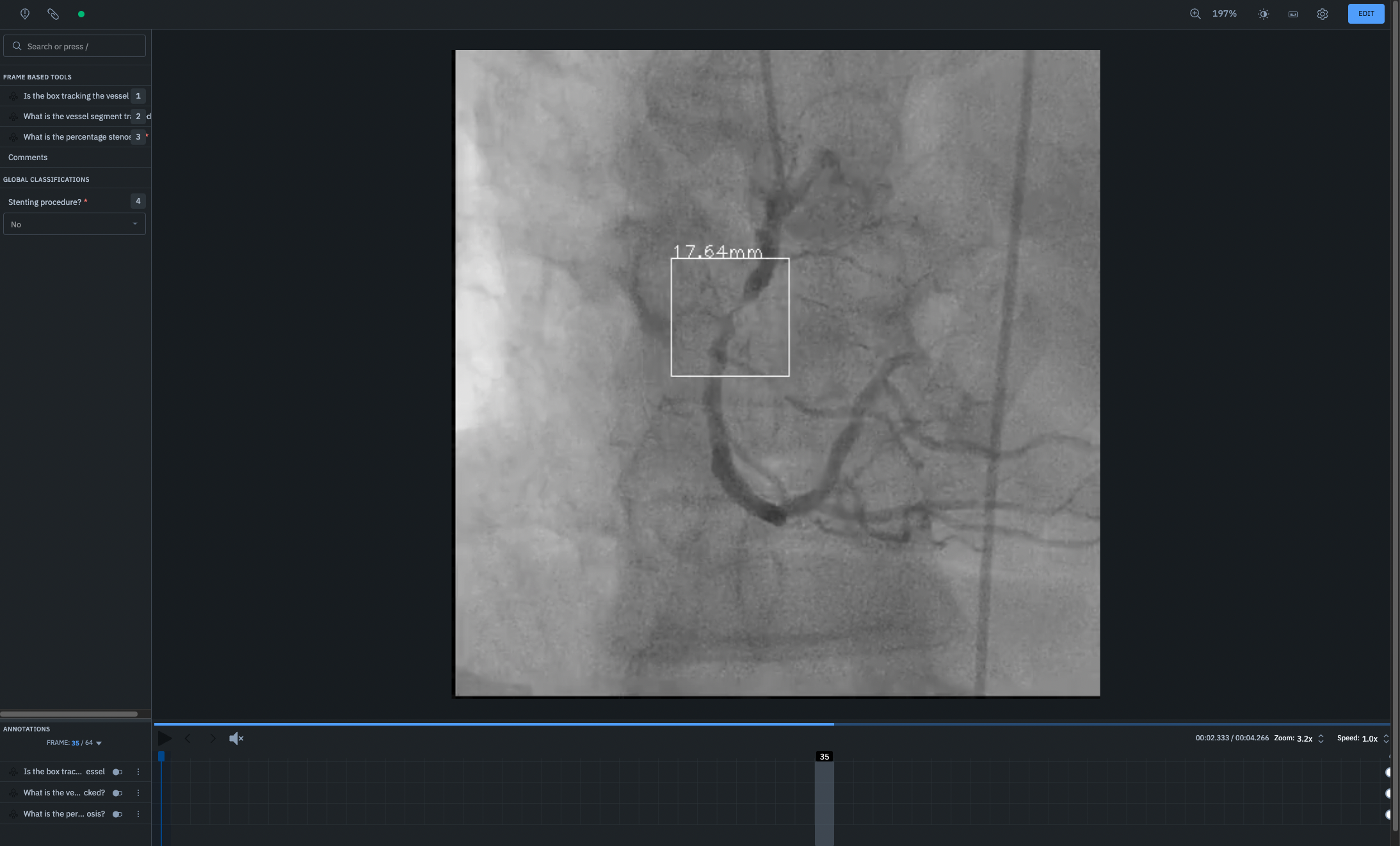
**

**Legend**. Interface which was used by the two cardiologists to annotate the percentage of stenosis, coronary artery segment being tracked, correctness of the registration and presence of a PCI in Dataset B. Annotations were made on labelbox.com.

**Supplementary Figure 3.** Scatterplot of DeepCoro’s Predictions as a Function of the Annotated Percentage Stenosis for (a) Dataset A, (b) Dataset B and (c) Dataset D

**Legend**. DeepCoro’s predictions at the video-level, in the Test Set, plotted against the annotated percentage stenosis, which is obtained with visual assessment from clinical reports for Dataset A, visual re-assessment for Dataset B and QCA for Dataset D. **Abbreviations**. QCA: Quantitative Coronary Angiography, *r*: Pearson’s correlation coefficient.

**Supplementary Figure 4.** DeepCoro’s Predictions as a Function of the Annotated Percentage Stenosis for (a) Dataset A, (b) Dataset B and (c) Dataset D Presented with Boxplots

**Legend**. DeepCoro’s predictions at the video-level presented as overlapping boxplots, in the Test Set, plotted against the annotated percentage stenosis, which is obtained with visual assessment from clinical reports for Dataset A, visual re-assessment for Dataset B and QCA for Dataset D. The intervals in part c. of the figure are established to ensure an equal separation of samples within each interval. **Boxplot centerline**: Median of the data. **Boxplot limits**: First quartile (25th percentile) and third quartile (75th percentile) of the data. **Boxplot whiskers**: Range of the data within 1.5 times of the quartiles. **Abbreviations**. QCA: Quantitative Coronary Angiography.

**Supplementary Figure 5**. Detailed datasets and patients size change.

**Legend**. Detailed datasets change in size when our algorithms are applied to our datasets. **Grey box**: Intermediate datasets. **Green box**: Final datasets. **Orange box**: Dataset split for the development of an algorithm. **Abbreviations**: ARCADE: Automatic Region-based Coronary Artery Disease diagnostics using X-ray angiography imagEs, CABG: Coronary Artery Bypass Grafting, CAG: Coronary Angiography, DICOM: Digital Imaging and Communications in Medicine, MHI: Montreal Heart Institute, PCI: Percutaneous Coronary Intervention, QCA: Quantitative Coronary Angiography.

**Supplementary Figure 6**. Summarisation of the number and fractions of sample that could not be processed due to technical limitations in the creation of Dataset A

**Legend**. Number of stenosis videos, DICOMs, patients and exams that could not be processed due to the intentional exclusion or limitations of DeepCoro in the creation of Dataset A. **Grey box**: Dataset size. **Purple box**: Exclusion box indicating the number of samples being removed. **Red text**: Samples removed. **Abbreviations**. DICOM: Digital Imaging and Communications in Medicine, CABG: Coronary Artery Bypass Grafting, PCI: Percutaneous Coronary Intervention.

**Supplementary Video 1**. Video Example of Outputs from the DeepCoro Pipeline


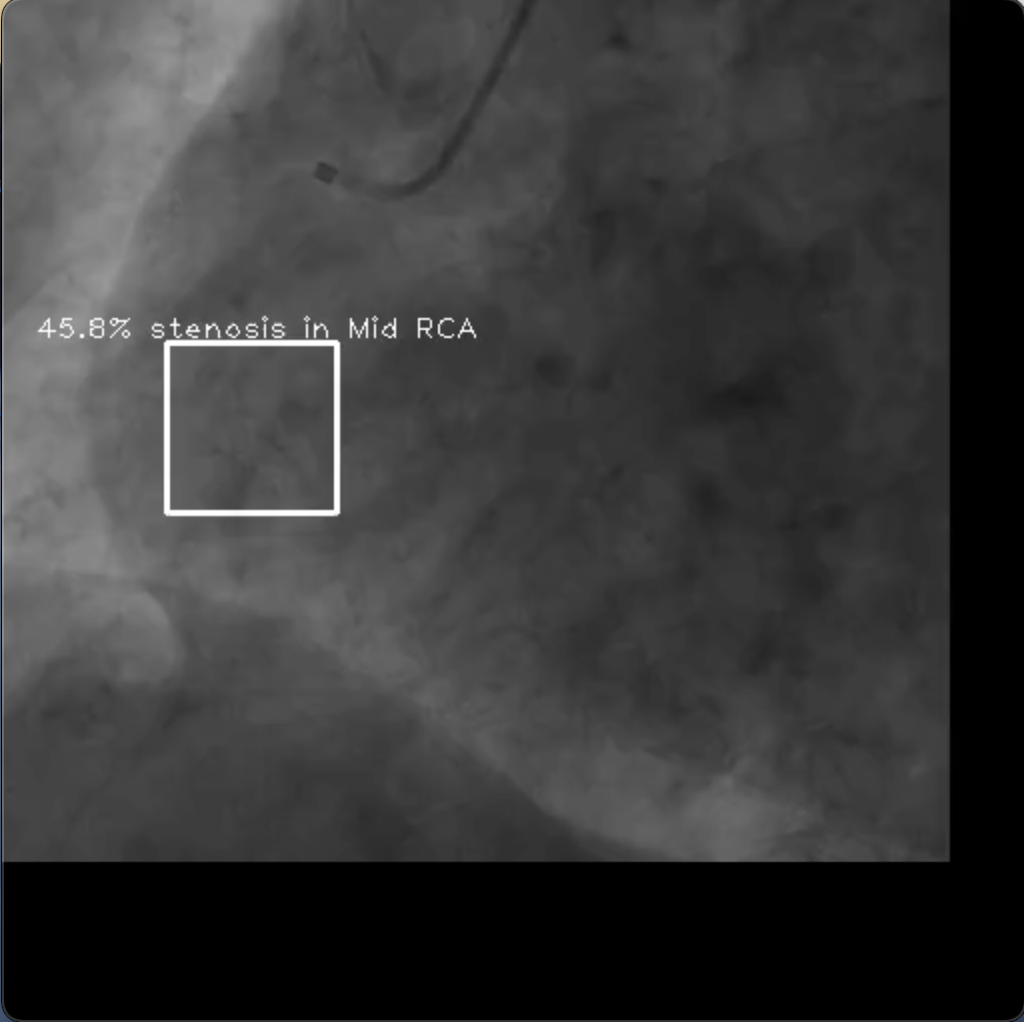


**Legend**. Video example of DeepCoro being applied to a video of the RCA with the outputs from all algorithms assembled. In the PDF version of this article, please click anywhere on the figure or caption to play the video in a separate window. **Abbreviations**. Mid RCA: middle right coronary artery, RCA: right coronary artery.

# Supplementary Tables

**Supplementary Table 1.** Baseline Characteristics of Datasets

| **Characteristic** | | | | **Dataset A** | **Dataset B** | **Dataset C** | **Dataset D** |
| --- | --- | --- | --- | --- | --- | --- | --- |
| **Patient information** | | | | | | | |
| Age (mean ± SD) | | | | 67.6 ± 11.0 | 67.6 ± 11.1 | Unspecified | 61.7 ± 9.0 |
| Sex | | | Female | 2,344 | 430 | 0 | 0 |
|  |  |  | Male | 5,532 | 1,162 | 0 | 0 |
|  |  |  | Unspecified | 181 | 36 | 1,200 | 1,010 |
| **Dataset information** | | | | | | | |
| Data type | | | | Video | Video | Image | Video |
| Number of videos or images | | | | 44,139 | 1,926 | 1,200 | 5,904 |
| Number of severe stenoses (n (%)) | | | | 7,076 (16%) | 431 (22%) | Unspecified | 493 (8%) |
| Number of severe non-stenoses (n (%)) | | | | 37,063 (84%) | 1,495 (78%) | Unspecified | 5,411 (92%) |
| Number of stenoses >0% (n (%)) | | | | 22,626 (51%) | 1,367 (71%) | Unspecified | 5,904 (100%) |
| Number of healthy vessels, 0% stenoses (n (%)) | | | | 21,513 (49%) | 559 (29%) | Unspecified | 0 (0%) |
| Average percentage of stenoses (mean ± SD) | | | | 20.6 ± 30.2 | 32.1 ± 31.3 | Unspecified | 33.7 ± 11.7 |
| Median percentage stenosis and interquartile range (median (Q1, Q2)) | | | | 10 (0, 30) | 20 (0, 60) | Unspecified | 31.3 (25.4, 39.0) |
| Number of videos for each artery | | | LCA | 21,892 | 1,011 | 759 | 2,595 |
|  |  |  | RCA | 22,247 | 915 | 441 | 3,309 |
| Number of patients | | | | 8,057 | 1,628 | Unspecified | 1,010 |
| Number of exams | | | | 8,524 | 1,653 | Unspecified | 1,325 |
| Number of videos or images per patient (mean ± SD) | | | | 5.5 ± 3.7 | 1.2 ± 0.5 | Unspecified | 5.8 ± 5.8 |
| Number of exams per patient (mean ± SD) | | | | 1.1 ± 0.3 | 1.0 ± 0.1 | Unspecified | 1.3 ± 1.5 |
| *Number of videos from exams in the corresponding artery segment* | | | | | | | |
| RCA | Single stenoses | | | 7,113 | 211 | Unspecified | 457 |
|  | Multiple stenoses | | | 12,410 | 638 | Unspecified | 2,852 |
| LCA | All | Single stenoses | | 4,621 | 174 | Unspecified | 230 |
|  |  | Multiple stenoses | | 16,158 | 806 | Unspecified | 2,365 |
|  | LCX | Single stenoses | | 11,178 | 513 | Unspecified | 961 |
|  |  | Multiple stenoses | | 2,992 | 149 | Unspecified | 803 |
|  | LAD | Single stenoses | | 11,305 | 506 | Unspecified | 622 |
|  |  | Multiple stenoses | | 7,928 | 432 | Unspecified | 1,831 |

**Legend**. Detailed table of the characteristics of each dataset. Severe stenoses are ≥70% for Datasets A and B, and ≥50% for Dataset D. **Abbreviations**. LCA: Left Coronary Artery, RCA: Right Coronary Artery, SD: Standard Deviation.

**Supplementary Table 2.** Baseline Characteristics of the Train, Validation, Derivation and Test Sets for Each Datasets

| **Characteristic** | | | **Dataset A** | | | **Dataset B** | | **Dataset C** | | **Dataset D** | |
| --- | --- | --- | --- | --- | --- | --- | --- | --- | --- | --- | --- |
| **Task** | | | Stenosis percentage prediction algorithm | | | PCI removal algorithm | | Segmentation algorithm | | Fine-tuning on the QCA labels | |
| **Split** | | | **Train** | **Validation** | **Test** | **Derivation** | **Test** | **Train and validation** | **Test** | **Train** | **Test** |
| **Patient information** | | | | | | | | | | | |
| Age (mean ± SD) | | | 67.4 ± 11.0 | 68.9 ± 10.6 | 67.5 ± 11.2 | 67.8 ± 11.2 | 66.9 ± 10.8 | Unspecified | Unspecified | 61.6 ± 9.0 | 62.0 ± 8.9 |
| Sex | Female | | 1,773 | 236 | 335 | 315 | 91 | 0 | 0 | 0 | 0 |
|  | Male | | 4,163 | 536 | 833 | 836 | 227 | 0 | 0 | 0 | 0 |
|  | Unspecified | | 131 | 23 | 27 | 29 | 7 | 1,000 | 200 | 825 | 206 |
| **Dataset information** | | | | | | | | | | | |
| Data type | | | Video | | | Video | | Image | | Video | |
| Number of videos or images | | | 32,629 | 4,608 | 6,902 | 1,335 | 333 | 1,000 | 200 | 4,637 | 1,267 |
| Number of severe stenoses (n (%)) | | | 4,802 (15%) | 912 (20%) | 1,353 (20%) | 300 (22%) | 71 (21%) | Unspecified | Unspecified | 341 (7%) | 152 (12%) |
| Number of severe non-stenoses (n (%)) | | | 27,827 (85%) | 3,696 (80%) | 5,549 (80%) | 1,005 (78%) | 262 (79%) | Unspecified | Unspecified | 4,296 (93%) | 1,115 (88%) |
| Number of stenoses >0% (n (%)) | | | 16,493 (51%) | 2,521 (55%) | 3,612 (52%) | 947 (71%) | 242 (73%) | Unspecified | Unspecified | 4,637 (100%) | 1,267 (100%) |
| Number of healthy vessels, 0% stenoses (n (%)) | | | 16,136 (49%) | 2,087 (45%) | 3,290 (48%) | 388 (29%) | 91 (27%) | Unspecified | Unspecified | 0 (0%) | 0 (0%) |
| Average percentage of stenoses (mean ± SD) | | | 19.6 ± 29.4 | 23.8 ± 32.2 | 23.2 ± 32.1 | 31.8 ± 31.1 | 32.8 ± 31.2 | Unspecified | Unspecified | 33.5 ± 11.6 | 34.4 ± 12.3 |
| Median percentage stenosis and interquartile range (median (Q1, Q2)) | | | 10 (0, 30) | 10 (0, 30) | 10 (0, 50) | 20 (0, 60) | 20 (0, 60) | Unspecified | Unspecified | 31.0 (25.5, 39.0) | 31.9 (25.4, 40.1) |
| Number of videos for each artery | LCA | | 16,208 | 2,268 | 3,416 | 694 | 175 | 625 | 134 | 2,039 | 556 |
|  | RCA | | 16,421 | 2,340 | 3,486 | 641 | 158 | 375 | 66 | 2,598 | 711 |
| Number of patients | | | 6,067 | 795 | 1,195 | 1,180 | 325 | Unspecified | Unspecified | 825 | 206 |
| Number of exams | | | 6,390 | 849 | 1,285 | 1,194 | 326 | Unspecified | Unspecified | 1,063 | 262 |
| Number of videos or images per patient (mean ± SD) | | | 5.4 ± 3.6 | 5.9 ± 3.7 | 5.9 ± 4.0 | 1.1 ± 0.4 | 1.0 ± 0.2 | Unspecified | Unspecified | 5.6 ± 5.4 | 6.2 ± 7.0 |
| Number of exams per patient (mean ± SD) | | | 1.1 ± 0.3 | 1.1 ± 0.3 | 1.1 ± 0.3 | 1.0 ± 0.1 | 1.0 ± 0.1 | Unspecified | Unspecified | 1.3 ± 0.5 | 1.3 ± 0.5 |
| *Number of videos from exams in the corresponding artery segment* | | | | | | | | | | | |
| RCA | Single stenoses | | 5,348 | 668 | 1,097 | 132 | 46 | Unspecified | Unspecified | 346 | 111 |
|  | Multiple stenoses | | 8,942 | 1,462 | 2,006 | 469 | 96 | Unspecified | Unspecified | 2,252 | 600 |
| LCA | All | Single stenoses | 3,601 | 429 | 591 | 121 | 33 | Unspecified | Unspecified | 198 | 32 |
|  |  | Multiple stenoses | 11,728 | 1,763 | 2,667 | 551 | 135 | Unspecified | Unspecified | 1,841 | 524 |
|  | LCX | Single stenoses | 8,158 | 1,228 | 1,792 | 349 | 90 | Unspecified | Unspecified | 748 | 213 |
|  |  | Multiple stenoses | 2,096 | 329 | 567 | 104 | 17 | Unspecified | Unspecified | 613 | 190 |
|  | LAD | Single stenoses | 8,431 | 1,135 | 1,739 | 350 | 84 | Unspecified | Unspecified | 491 | 131 |
|  |  | Multiple stenoses | 5,743 | 880 | 1,305 | 289 | 79 | Unspecified | Unspecified | 1,429 | 402 |

**Legend**. Detailed table of the characteristics of each split of each dataset. Severe stenoses are ≥70% for Datasets A and B, and ≥50% for Dataset D. **Abbreviations**. LCA: Left Coronary Artery, RCA: Right Coronary Artery, SD: Standard Deviation

**Supplementary Table 3**. Possible Class Outputs of Algorithm 1, Algorithm 2 and Algorithm 4 and Their Definitions

| **Algorithm** | **Possible class outputs** | **Definitions** |
| --- | --- | --- |
| Primary anatomic structure identification  Algorithm  (Algorithm 1) ^1^ | Aorta | “Ascending aorta, the arch or descending aorta, as delimited during aortography.” ^1^ |
|  | Catheter | “Any guiding catheter or diagnostic catheter without any other underlying structure.” ^1^ |
|  | Femoral artery | “Either the superficial, deep or common femoral artery.” ^1^ |
|  | Bypass graft | “Venous graft, internal mammary graft or radial graft.” ^1^ |
|  | Left ventricle | “Ventricle, as delimited during ventriculography” ^1^ |
|  | Left coronary artery | “Artery that arises from the aorta above the left cusp of the aortic valve” ^1^ |
|  | Other | “Any images not belonging to the other classes (for example, kidneys, pacemaker, etc) ” ^1^ |
|  | Pigtail catheter | “Pigtail catheter without any other underlying structure” ^1^ |
|  | Radial artery | “Major artery in the forearm” ^1^ |
|  | Right coronary artery | “Artery that arises from the aorta above the right cusp of the aortic valve” ^1^ |
|  | Stenting procedure | Stenting procedure |
| Stenosis detection algorithm (Algorithm 2) ^1^ | Proximal right coronary artery | “From ostium to one half the distance to the acute margin of the heart. ” ^1,2^ |
|  | Middle right coronary artery | “From end of first segment to acute margin of heart. ” ^1,2^ |
|  | Distal right coronary artery | “From the acute margin of the heart to the origin of the posterior descending artery. ” ^1,2^ |
|  | Posterior descending artery | “Artery running the posterior interventricular groove. ” ^1,2^ |
|  | Posterolateral branch from the right coronary artery | “Posterolateral branch originating from the distal coronary artery distal to the crux. If left posterolateral, it was chosen as the artery running to the posterolateral surface of the left ventricle. ” ^1,2^ |
|  | Left main artery | “From the ostium of the LCA through bifurcation into left anterior descending and left circumflex branches. ” ^1,2^ |
|  | Proximal left anterior descending artery | “Vessel between left main and proximal to and including the first septal” ^1,2^ |
|  | Middle left anterior descending artery | “LAD immediately distal to the origin of first septal branch and extending to the point where the LAD forms an angle (right anterior oblique projection). If angle is not identifiable, this segment ends at one half the distance form the first septal and the apex of the heart” ^1,2^ |
|  | Distal left anterior descending artery | “Terminal portion of LAD, beginning at the end of previous segment and extending to or beyond the apex. ” ^1,2^ |
|  | Proximal left circumflex artery | “Main stem of circumflex from its origin of left main to and including origin of first obtuse marginal branch. ” ^1,2^ |
|  | Distal left circumflex artery | “The stem of the circumflex distal to the origin of the most distal obtuse marginal branch and running along the posterior left atrioventricular grooves. Caliber may be small or artery absent. ” ^1,2^ |
|  | Valve | “Presence of a mechanical valve, annuloplasty or valvular calcifications” ^1,2^ |
|  | Catheter | “Presence of a catheter, such as a diagnostic catheter, pigtail or guiding catheter” ^1,2^ |
|  | Sternotomy | “Presence of sternotomy wires” ^1,2^ |
|  | Stent | “Stent landmarks on a guidewire or in a vessel” ^1,2^ |
|  | Pacemaker | “Presence of a pacemaker or pacemaker lead” ^1,2^ |
|  | Guidewire | “Presence of a guide wire” ^1,2^ |
|  | **Stenosis*** | “Any visible stenosis” ^1,2^ |
|  | Obstruction | “100% obstruction of an artery, either by thrombus or chronically occluded. Defined by a blunt stump at the end to a vessel or by the ‘absence’ of contrast in between two healthy vessel segments with bridging collaterals. ” ^1,2^ |
| Segmentation algorithm (Algorithm 4) ^2,3^ | Proximal right coronary artery (1) | “From the ostium to one half the distance to the acute margin of the heart.” ^2^ |
|  | Middle right coronary artery (2) | “From the end of first segment to acute margin of heart. ” ^2^ |
|  | Distal right coronary artery (3) | “From the acute margin of the heart to the origin of the posterior descending artery. ” ^2^ |
|  | Posterior descending artery (4) | “Running in the posterior interventricular groove” ^2^ |
|  | Left main artery (5) | “From the ostium of the LCA through bifurcation into left anterior descending and left circumflex branches. ” ^2^ |
|  | Proximal left anterior descending artery (6) | “Proximal to and including first major septal branch.” ^2^ |
|  | Middle left anterior descending artery (7) | “LAD immediately distal to origin of first septal branch and extending to the point where LAD forms an angle (right anterior oblique view). If this angle is not identifiable this segment ends at one half the distance from the first septal to the apex of the heart. ” ^2^ |
|  | Distal (apical) left anterior descending artery (8) | “Terminal portion of LAD, beginning at the end of previous segment and extending to or beyond the apex” ^2^ |
|  | First diagonal (9) | “The first diagonal originating from segment 6 or 7. ” ^2^ |
|  | First diagonal a (9a) | “Additional first diagonal originating from segment 6 or 7, before segment 8. ” ^2^ |
|  | Second diagonal (10) | “Originating from segment 8 or the transition between segment 7 and 8. ” ^2^ |
|  | Second diagonal a (10a) | “Additional second diagonal originating from segment 8. ” ^2^ |
|  | Proximal left circumflex artery (11) | “Main stem of circumflex from its origin of left main and including origin of first obtuse marginal branch” ^2^ |
|  | Intermediate/anterolateral (12) | “Branch from trifurcating left main other than proximal LAD or LCX. It belongs to the circumflex territory. ” ^2^ |
|  | Obtuse marginal a (12a) | “First side branch of circumflex running in general to the area of obtuse margin of the heart. ” ^2^ |
|  | Distal left circumflex artery (13) | “The stem of the circumflex distal to the origin of the most distal obtuse marginal branch, and running along the posterior left atrioventricular groove. Caliber may be small or artery absent. ” ^2^ |
|  | Left posterolateral (14) | “Running to the posterolateral surface of the left ventricle. May be absent or a division of obtuse marginal branch. ” ^2^ |
|  | Left posterolateral a (14a) | “Distal from 14 and running in the same direction.” ^2^ |
|  | Posterior descending artery (15) | “Most distal part of dominant left circumflex when present. It gives origin to septal branches. When this artery is present, segment 4 is usually absent. ” ^2^ |
|  | Posterolateral branch from the right coronary artery (16) | “Posterolateral branch originating from the distal coronary artery distal to the crux. ” ^2^ |
|  | Posterolateral branch from the right coronary artery a (16a) | “First posterolateral branch from segment 16. ” ^2^ |
|  | Posterolateral branch from the right coronary artery b (16b) | “Second posterolateral branch from segment 16. ” ^2^ |
|  | Posterolateral branch from the right coronary artery c (16c) | “Third posterolateral branch from segment 16. ” ^2^ |
|  | Obtuse marginal b (12b) | “Second additional branch of circumflex running in the same direction as 12” ^2^ |
|  | Left posterolateral b (14b) | “Distal from 14 and 14 a and running in the same direction. ” ^2^ |

**Legend**. Listing of the classes and definitions of our different multi-class algorithms. **Asterix**: Only this class was pertinent for for DeepCORO, the other classes are legacy and deprecated, they belong to previous work on CathAI. **Abbreviations**. LAD: Left Anterior Descending Artery, LCA: Left Coronary Artery, LCX: Left Circumflex.

**Supplementary Table 4**. Segmentation Performance of DeepCoro’s Algorithm 4 on the Test Set of Dataset C

| **Coronary artery** | **Coronary artery segment** | **Number of instances** | **Dice Score (%)** | **PPV (%)** | **Sensitivity (%)** |
| --- | --- | --- | --- | --- | --- |
| LCA | left main artery | 129 | 77.84 | 86.48 | 70.77 |
|  | proximal left anterior descending artery | 108 | 70.89 | 68.70 | 73.22 |
|  | middle left anterior descending artery | 73 | 74.20 | 76.53 | 72.01 |
|  | distal left anterior descending artery | 71 | 71.40 | 74.13 | 68.87 |
|  | proximal left circumflex artery | 67 | 75.14 | 74.35 | 75.96 |
|  | distal left circumflex artery | 62 | 60.39 | 61.15 | 59.66 |
| RCA | proximal right coronary artery | 65 | 80.33 | 80.92 | 79.76 |
|  | middle right coronary artery | 66 | 72.58 | 78.87 | 67.22 |
|  | distal right coronary artery | 66 | 77.26 | 76.92 | 77.60 |
|  | posterolateral branch from the right coronary artery | 49 | 63.08 | 82.44 | 51.09 |
|  | posterior descending artery | 48 | 74.11 | 77.02 | 71.42 |
| weighted average | | | 72.93 | 75.96 | 70.12 |

**Legend**. Detailed performance of DeepCoro’s Algorithm 4 on the test set of Dataset C across coronary artery segments. **Abbreviations**. LCA: Left Coronary Artery, PPV: Positive Predictive Value, RCA: Right Coronary Artery.

**Supplementary Table 5**.  Comparative Performance of DeepCoro's Segmentation and CathAI's Bounding Box Method for Stenosis Assignment to Coronary Segments in Dataset B

| **Coronary artery** | **Artery segment** | **Number of videos** | **Metric** | | | | | |
| --- | --- | --- | --- | --- | --- | --- | --- | --- |
|  |  |  | ***PPV (%)*** | | ***Sensitivity (%)*** | | ***F1-score (%)*** | |
|  |  |  | ***RetinaNet*** | ***Segmentation*** | ***RetinaNet*** | ***Segmentation*** | ***RetinaNet*** | ***Segmentation*** |
| LCA | left main artery | 129 | 47.09 | **81.65** | **75.19** | 68.99 | 57.91 | **74.79** |
|  | proximal left anterior descending artery | 207 | 40.74 | **52.79** | 53.14 | **77.78** | 46.12 | **62.89** |
|  | middle left anterior descending artery | 207 | 54.76 | **63.69** | 33.33 | **51.69** | 41.44 | **57.07** |
|  | distal left anterior descending artery | 91 | 65.06 | **85.71** | 59.34 | **65.93** | 62.07 | **74.53** |
|  | proximal left circumflex artery | 204 | 49.22 | **72.89** | 46.57 | **59.31** | 47.86 | **65.41** |
|  | distal left circumflex artery | 62 | 37.50 | **43.42** | 9.68 | **53.23** | 15.38 | **47.83** |
| RCA | proximal right coronary artery | 281 | 77.42 | **82.57** | 76.87 | **89.32** | 77.14 | **85.81** |
|  | middle right coronary artery | 361 | 76.56 | **78.69** | 67.87 | **79.78** | 71.95 | **79.23** |
|  | distal right coronary artery | 185 | 59.57 | **71.59** | 45.41 | **68.11** | 51.53 | **69.81** |
|  | posterolateral branch from the right coronary artery | 29 | 19.10 | **53.85** | **58.62** | 48.28 | 28.81 | **50.91** |
|  | posterior descending artery | 51 | 33.33 | **68.29** | 54.90 | 54.90 | 41.48 | **60.87** |
| weighted average | | | 59.10 | **71.89** | 56.50 | **70.72** | 56.50 | **70.71** |

**Legend**. Comparative table of the coronary artery segment prediction algorithm from CathAI and DeepCoro. RetinaNet corresponds to CathAI’s method to identify coronary artery segments and segmentation referrers to DeepCoro’s method. The statistically significant metrics where the confidence intervals don’t overlap are shown in bold. **Abbreviations**, LCA: Left Coronary Artery, PPV: Positive Predictive Value, RCA: Right Coronary Artery.

**Supplementary Table 6**. Artery-Level Performance of CathAI on the Test Set of Dataset A and Comparison to DeepCoro

| **Task** | **Metric** | **Coronary artery** | | | | | |
| --- | --- | --- | --- | --- | --- | --- | --- |
|  |  | ***LCA*** | | ***RCA*** | | ***RCA + LCA*** | |
|  |  | ***Image-based model*** | ***Video-based model*** | ***Image-based model*** | ***Video-based model*** | ***Image-based model*** | ***Video-based model*** |
| Number of exams | | 2568 | | 2259 | | 4827 | |
| Number of severe stenoses, ≥ 70% | | 536 | | 345 | | 881 | |
| Number of healthy vessels, 0% stenoses | | 1253 | | 1075 | | 2328 | |
| Classification | AUROC | 0.7418 (0.7303 - 0.7526) | **0.8017 (0.7919 - 0.8124)** | 0.8561 (0.8455 - 0.8682) | 0.8643 (0.8537 - 0.8745) | 0.7953 (0.7875 - 0.8038) | **0.8294 (0.8215 - 0.8373)** |
|  | AUPRC | 0.4235 (0.4029 - 0.4429) | **0.5092 (0.4868 - 0.5329)** | 0.5312 (0.5008 - 0.5619) | 0.5578 (0.5242 - 0.5890) | 0.4670 (0.4497 - 0.4849) | **0.5239 (0.5041 - 0.5421)** |
|  | Sensitivity (%) | 61.01 (59.00 - 63.05) | **70.70 (68.75 - 72.73)** | 78.02 (75.87 - 80.28) | 76.20 (73.98 - 78.60) | 67.64 (66.09 - 69.31) | **72.86 (71.24 - 74.47)** |
|  | Specificity (%) | 74.61 (73.68 - 75.55) | 74.51 (73.56 - 75.43) | 80.75 (79.86 - 81.57) | 79.03 (78.10 - 80.04) | 77.57 (76.92 - 78.22) | 76.71 (76.05 - 77.36) |
|  | PPV (%) | 38.78 (37.15 - 40.32) | 41.06 (39.48 - 42.70) | **42.25 (40.23 - 44.11)** | 37.08 (35.11 - 39.00) | 40.25 (38.97 - 41.55) | 39.42 (38.15 - 40.68) |
|  | F1-score (%) | 47.41 (45.81 - 48.96) | **51.95 (50.32 - 53.58)** | **54.81 (52.81 - 56.53)** | 49.88 (47.86 - 51.78) | 50.46 (49.23 - 51.72) | 51.15 (49.81 - 52.39) |
| Regression | MAE (%) | 23.81 (23.42 - 24.22) | **22.19 (21.82 - 22.52)** | 19.11 (18.76 - 19.46) | **17.82 (17.48 - 18.16)** | 21.61 (21.35 - 21.87) | **20.15 (19.88 - 20.40)** |
|  | *r* | 0.3704 (0.3520 - 0.3880) | **0.4890 (0.4704 - 0.5087)** | 0.5554 (0.5349 - 0.5770) | **0.6200 (0.6018 - 0.6372)** | 0.4571 (0.4430 - 0.4711) | **0.5497 (0.5360 - 0.5630)** |

**Legend**. Comparative table of the artery-level performance of the percentage of stenosis prediction algorithm from CathAI and DeepCoro. The image-based refers to the retrained classifier from CathAI and the video-based refers to DeepCoro Algorithm 6. The statistically significant metrics where the confidence intervals don’t overlap are shown in bold. DeepCoro and CathAI predictions were binarized with a threshold of 0.23 and 0.22 respectively, as determined on the validation set. The range in parentheses is the 95% confidence interval generated by bootstrapping. **Abbreviations**. AUPRC: Area Under the Precision-Recall Curve, AUROC: Area Under the Receiver Operating Curve, LCA: Left Coronary Artery, MAE: Mean Absolute Error, PPV: Positive Predictive Value, *r*: Pearson’s correlation coefficient, RCA: Right Coronary Artery.

**Supplementary Table 7**. Video-Level Performance of CathAI on the Test Set of Dataset A and Comparison to DeepCoro

| **Task** | **Metric** | **Coronary artery** | | | | | |
| --- | --- | --- | --- | --- | --- | --- | --- |
|  |  | ***LCA*** | | ***RCA*** | | ***RCA + LCA*** | |
|  |  | ***Image-based model*** | ***Video-based model*** | ***Image-based model*** | ***Video-based model*** | ***Image-based model*** | ***Video-based model*** |
| Number of videos | | 3416 | | 3486 | | 6902 | |
| Number of severe stenoses, ≥ 70% | | 776 | | 577 | | 1353 | |
| Number of healthy vessels, 0% stenoses | | 1782 | | 1830 | | 3612 | |
| Classification | AUROC | 0.7197 (0.7099 - 0.7292) | **0.7798 (0.7713 - 0.7886)** | 0.8355 (0.8265 - 0.8452) | 0.8463 (0.8378 - 0.8552) | 0.7767 (0.7700 - 0.7838) | **0.8114 (0.8052 - 0.8177)** |
|  | AUPRC | 0.4266 (0.4095 - 0.4449) | **0.5220 (0.5039 - 0.5415)** | 0.5132 (0.4911 - 0.5350) | **0.5776 (0.5547 - 0.5989)** | 0.4637 (0.4498 - 0.4780) | **0.5428 (0.5279 - 0.5586)** |
|  | Sensitivity (%) | 58.39 (56.68 - 60.16) | **67.15 (65.54 - 68.89)** | 76.28 (74.67 - 78.02) | 77.31 (75.65 - 79.10) | 65.98 (64.78 - 67.21) | **71.45 (70.31 - 72.74)** |
|  | Specificity (%) | 73.03 (72.20 - 73.85) | 73.53 (72.63 - 74.41) | 78.30 (77.53 - 79.02) | 77.37 (76.61 - 78.12) | 75.77 (75.22 - 76.36) | 75.55 (75.02 - 76.13) |
|  | PPV (%) | 38.88 (37.46 - 40.26) | **42.74 (41.40 - 44.15)** | 41.06 (39.54 - 42.64) | 40.38 (38.93 - 41.85) | 39.87 (38.94 - 40.82) | 41.61 (40.54 - 42.56) |
|  | F1-score (%) | 46.67 (45.31 - 48.00) | **52.23 (50.89 - 53.50)** | 53.38 (51.89 - 54.83) | 53.05 (51.63 - 54.53) | 49.71 (48.77 - 50.63) | **52.59 (51.57 - 53.59)** |
| Regression | MAE (%) | 24.60 (24.26 - 24.93) | **22.97 (22.66 - 23.29)** | 19.60 (19.32 - 19.87) | **18.25 (17.96 - 18.51)** | 22.07 (21.86 - 22.30) | **20.59 (20.38 - 20.80)** |
|  | *r* | 0.3470 (0.3312 - 0.3631) | **0.4624 (0.4476 - 0.4771)** | 0.5389 (0.5223 - 0.5553) | **0.6027 (0.5878 - 0.6179)** | 0.4432 (0.4321 - 0.4545) | **0.5312 (0.5210 - 0.5423)** |

**Legend**. Comparative table of the video-level performance of the percentage of stenosis prediction algorithm from CathAI and DeepCoro. The image-based refers to the retrained classifier from CathAI and the video-based refers to DeepCoro Algorithm 6. The statistically significant metrics where the confidence intervals don’t overlap are shown in bold. DeepCoro and CathAI predictions were binarized with a threshold of 0.23 and 0.22 respectively, as determined on the validation set. The range in parentheses is the 95% confidence interval generated by bootstrapping. **Abbreviations**. AUPRC: Area Under the Precision-Recall Curve, AUROC: Area Under the Receiver Operating Curve, LCA: Left Coronary Artery, MAE: Mean Absolute Error, PPV: Positive Predictive Value, *r*: Pearson’s correlation coefficient, RCA: Right Coronary Artery.

**Supplementary Table 8**. Performance of DeepCoro at the Arterial Level on Dataset A's Test Set, Segregated by Age and Sex

| Category | Number of exams | Number of severe stenoses | Classification | | | | Regression | |
| --- | --- | --- | --- | --- | --- | --- | --- | --- |
|  |  |  | AUROC | AUPRC | Sensitivity (%) | Specificity (%) | MAE (%) | *r* |
| **Sexes** | | | | | | | | |
| Female | 2655 | 605 | 0.8420 (0.8283 - 0.8564) | 0.4950 (0.4565 - 0.5350) | 72.29 (69.23 - 75.48) | 77.51 (76.27 - 78.81) | 19.13 (18.62 - 19.59) | 0.5649 (0.5392 - 0.5907) |
| Male | 1069 | 198 | 0.8203 (0.8115 - 0.8295) | 0.5260 (0.5055 - 0.5491) | 72.30 (70.62 - 74.06) | 76.25 (75.40 - 77.05) | 20.53 (20.20 - 20.83) | 0.5438 (0.5284 - 0.5596) |
| **Age groups** | | | | | | | | |
| < 60 | 1127 | 155 | 0.8549 (0.8400 - 0.8729) | 0.5747 (0.5331 - 0.6197) | 67.72 (64.17 - 71.54) | 83.83 (82.82 - 84.94) | 17.98 (17.51 - 18.47) | 0.5696 (0.5431 - 0.5970) |
| ≥ 60 and < 67 | 1030 | 175 | 0.8107 (0.7943 - 0.8281) | 0.5116 (0.4745 - 0.5508) | 69.10 (65.54 - 72.66) | 78.34 (76.99 - 79.71) | 19.62 (19.06 - 20.14) | 0.5425 (0.5144 - 0.5702) |
| ≥ 67 and < 75 | 1233 | 213 | 0.8064 (0.7919 - 0.8216) | 0.4523 (0.4163 - 0.4841) | 71.82 (68.82 - 75.14) | 73.53 (72.22 - 74.82) | 20.64 (20.12 - 21.15) | 0.5123 (0.4868 - 0.5382) |
| ≥ 75 | 1369 | 271 | 0.8308 (0.8180 - 0.8440) | 0.5580 (0.5244 - 0.5911) | 77.48 (75.24 - 79.91) | 72.57 (71.23 - 73.98) | 21.54 (21.07 - 21.97) | 0.5646 (0.5429 - 0.5885) |

**Legend**. DeepCoro’s performance stratified across ages and sexes. The range in parentheses is the 95% confidence interval generated by bootstrapping. **Abbreviations**. AUPRC: Area Under the Precision-Recall Curve, AUROC: Area Under the Receiver Operating Curve, MAE: Mean Absolute Error, PPV: Positive Predictive Value, *r*: Pearson’s correlation coefficient.

**Supplementary Table 9**. Video-level and Artery-level Performance DeepCoro on Dataset D

| **Metrics** | **Coronary artery** | | |
| --- | --- | --- | --- |
|  | ***LCA*** | ***RCA*** | ***RCA + LCA*** |
| **Artery-level** | | | |
| Number of exams | 310 | 319 | 629 |
| MAE (%) | 8.18 (7.71 - 8.59) | 7.31 (6.82 - 7.72) | 7.75 (7.37 - 8.07) |
| *r* | 0.2858 (0.1997 - 0.3609) | 0.3899 (0.3353 - 0.4492) | 0.3439 (0.2970 - 0.3898) |
| **Video-level** | | | |
| Number of videos | 568 | 699 | 1267 |
| MAE (%) | 8.43 (8.11 - 8.75) | 8.43 (8.11 - 8.75) | 8.43 (8.21 - 8.64) |
| *r* | 0.2688 (0.2222 - 0.3146) | 0.3276 (0.2866 - 0.3683) | 0.3090 (0.2779 - 0.3396) |

**Legend**. DeepCoro’s performance when fine-tuned on QCA labels. The range in parentheses is the 95% confidence interval generated by bootstrapping. **Abbreviations**. LCA: Left Coronary Artery, MAE: Mean Absolute Error, QCA: Quantitative Coronary Angiography, *r*: Pearson’s correlation coefficient, RCA: Right Coronary Artery.

**Supplementary Table 10**. Parameters and Validation Set of Dataset C Dice Coefficient of the Seven Selected Models as Part of Algorithm 4

| **Training parameters** | **Dice coefficient on the validation set** |
| --- | --- |
| Model = FPN  Loss function = Lovasz Loss  Batch size = 64  Learning rate = 0.00107809 | 0.6730 |
| Model = DeepLabV3+  Loss function = Lovasz Loss  Batch size = 64  Learning rate = 0.00242160 | 0.6816 |
| Model = PAN  Loss function = Tversky Loss  Batch size = 64  Learning rate = 0.00129894 | 0.6744 |
| Model = DeepLabV3  Loss function = Tversky Loss  Batch size = 4  Learning rate = 0.00059902 | 0.6294 |
| Model = FPN  Loss function = Lovasz Loss  Batch size = 16  Learning rate = 0.00993245 | 0.6287 |
| Model = DeepLabV3  Loss function = Lovasz Loss  Batch size = 16  Learning rate = 0.00232125 | 0.6225 |
| Model = PAN  Loss function = Dice Loss  Batch size = 64  Learning rate = 0.00646546 | 0.6683 |

**Legend**. Parameters used for training the several selected segmentation model and the validation set Dice coefficient that allowed them to be selected. The Dice Coefficient here was calculated over the 25 coronary artery segments available in the ARCADE dataset.

**Supplementary Table 11**. Video-based model trainings for stenosis severity (Algorithm 6) as part of DeepCoro’s pipeline.

| **Training description** | **Training parameters** | **Best valdiation loss** | **Validation AUC associated to the best loss** |
| --- | --- | --- | --- |
| Swin3D (B) training for regression on Dataset A | LR = 1e-3  Loss = Mean square error  Model = Swin3D (B) | 0.07432 | 0.8143 |
| Swin3D (B) training for regression on Dataset A | LR = 1e-4  Loss = Mean square error  Model = Swin3D (B) | 0.07724 | 0.8085 |
| Swin3D (B) training for regression on Dataset A | LR = 1e-5  Loss = Mean square error  Model = Swin3D (B) | 0.08225 | 0.7789 |
| Swin3D (B) training for regression on Dataset A without adding age and artery segments in the last feature layer using different parameters for PCI cleaning (v2) and RetinaNet for coronary artery assignment | LR = 1e-4  Loss = Mean square error  Model = Swin3D (B) | 0.07305 | 0.7549 |
| SlowFast training for regression on Dataset A without adding age and artery segments in the last feature layer using different parameters for PCI cleaning (v2) and RetinaNet for coronary artery assignment | LR = 1e-4  Loss = Mean square error  Model = SlowFast (R101) | 0.07627 | 0.7237 |
| X3D (L) training for regression on Dataset A using RetinaNet for coronary artery assignment, and without adding age and artery segments in the last feature layer using different parameters for PCI cleaning (v2) | LR = 1e-4  Loss = Mean square error  Model = X3D (L) | 0.07337 | 0.7281 |
| Swin3D (B) training for classification on Dataset A using RetinaNet for coronary artery assignment, and without adding age and artery segments in the last feature layer using different parameters for PCI cleaning (v2) | LR = 1e-4  Loss = Cross entropy  Model = Swin3D (B) | 0.5384 | 0.7658 |
| Swin3D (S) training for classification on Dataset A using RetinaNet for coronary artery assignment, and without adding age and artery segments in the last feature layer using different parameters for PCI cleaning (v2) | LR = 1e-4  Loss = Cross entropy  Model = Swin3D (S) | 0.5483 | 0.7422 |
| Swin3D (S) training for classification on Dataset A using RetinaNet for coronary artery assignment, and without adding age and artery segments in the last feature layer using different parameters for PCI cleaning (v2) | LR = 1e-3  Loss = Cross entropy  Model = Swin3D (S) | 0.5209 | 0.7639 |
| Swin3D (S) training for classification on Dataset A using RetinaNet for coronary artery assignment, and without adding age and artery segments in the last feature layer using different parameters for PCI cleaning (v1) | LR = 1e-3  Loss = Cross entropy  Model = Swin3D (S) | 0.5519 | 0.7390 |
| MViT training for classification on Dataset A (2017, 2018, 2019) using RetinaNet for coronary artery assignment, and without adding age, removal of CAGB and artery segments in the last feature layer using different parameters for PCI cleaning (v1) | LR = 1e-2  Loss = Cross entropy  Model = MviT | 0.5423 | 0.6423 |
| X3D (L) training for classification on Dataset A (2017, 2018, 2019) using RetinaNet for coronary artery assignment, and without adding age, removal of CAGB and artery segments in the last feature layer using different parameters for PCI cleaning (v1) | LR = 1e-2  Loss = Cross entropy  Model = X3D (L) | 0.5151 | 0.6889 |
| X3D (M) training for classification on Dataset A (2017, 2018, 2019) using RetinaNet for coronary artery assignment, and without adding age, removal of CAGB and artery segments in the last feature layer using different parameters for PCI cleaning (v1) | LR = 1e-2  Loss = Cross entropy  Model = X3D (M) | 0.5244 | 0.6678 |
| R(2+1)D training for classification on Dataset A (2017, 2018, 2019) using RetinaNet for coronary artery assignment, and without adding age, removal of CAGB and artery segments in the last feature layer using different parameters for PCI cleaning (v1) | LR = 1e-2  Loss = Cross entropy  Model = R(2+1)D | 0.5505 | 0.5334 |

**Legend**. Various examples of trainings performed as part of the development process of DeepCoro for stenosis assessment. **Abbreviations**. CABG: Coronary Artery Bypass Grafting, LR: Learning Rate, PCI: Percutaneous Coronary Intervention.

**Supplementary Table 12**. Examples of CathAI’s vs. DeepCoro’s Approach for Coronary Artery Segment Assignment

| **Case** | **CathAI** | **DeepCoro** |
| --- | --- | --- |
| **RCA** | | |
| DeepCoro’s output and CathAI’s output are correct *(Ground truth: Prox RCA)* | 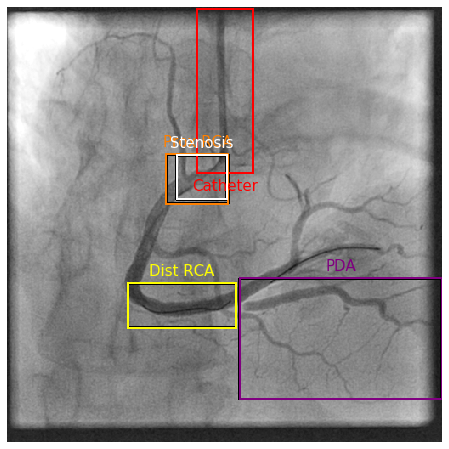  **Output**: Prox RCA | 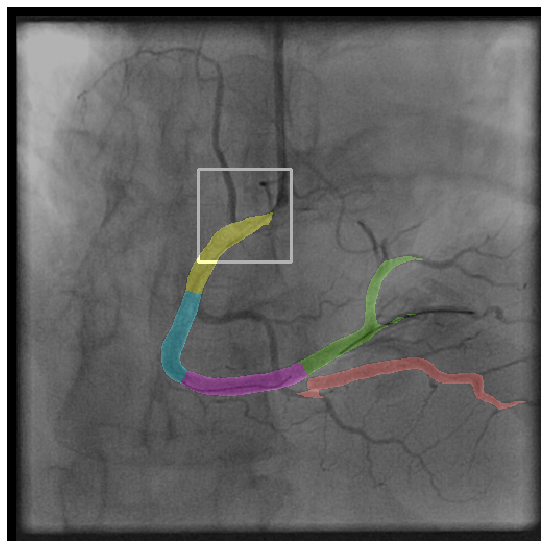  **Output**: Prox RCA |
| DeepCoro’s output is correct and CathAI’s output is incorrect *(Ground truth: Mid RCA)* | 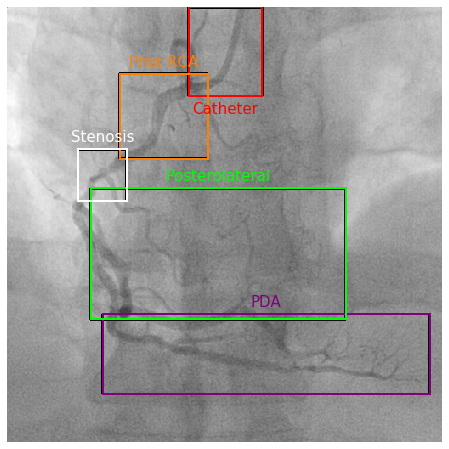  **Output**: Posterolateral  (No Mid RCA bounding boxes were detected, and the stenosis box overlaps primarily with a Posterolateral box.) | 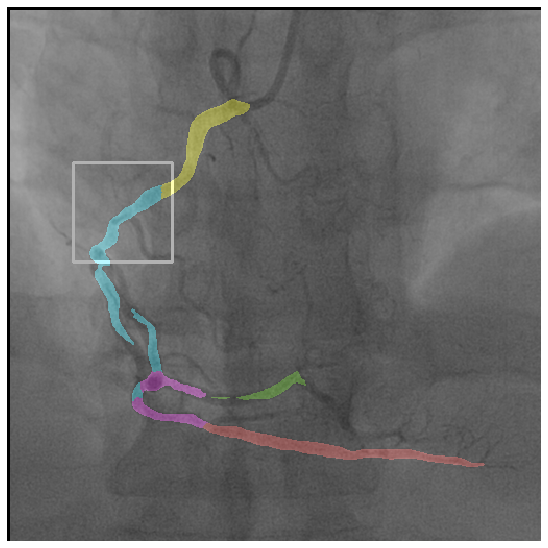  **Output**: Mid RCA |
| DeepCoro’s output is incorrect and CathAI’s output is correct *(Ground truth: Mid RCA)* | 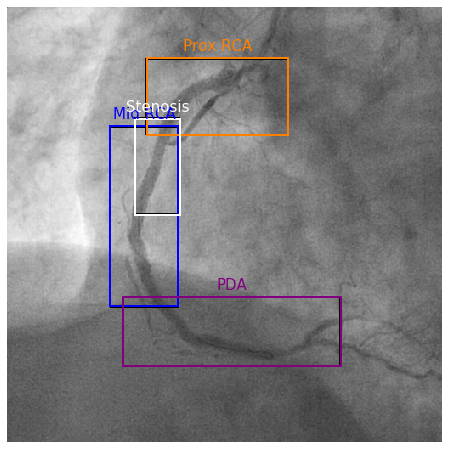  **Output**: Mid RCA | 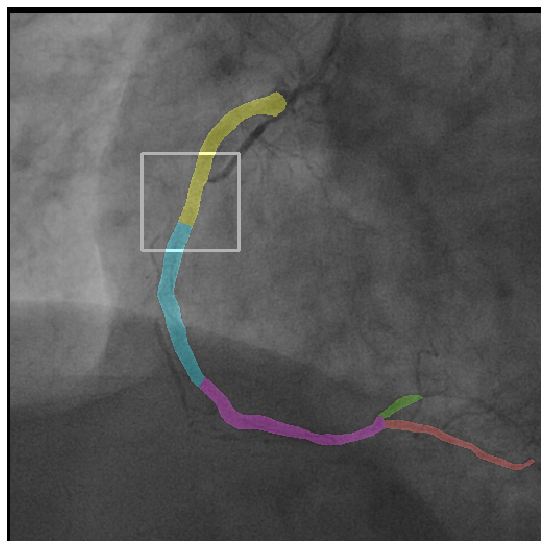  **Output**: Prox RCA  (The stenosis is located at the junction of the Prox RCA and Mid RCA segments. While the human operator identified the stenosis in Mid RCA, DeepCoro mistakenly assigned it to Prox RCA.) |
| DeepCoro’s output and CathAI’s output are incorrect *(Ground truth: Posterolateral)* | 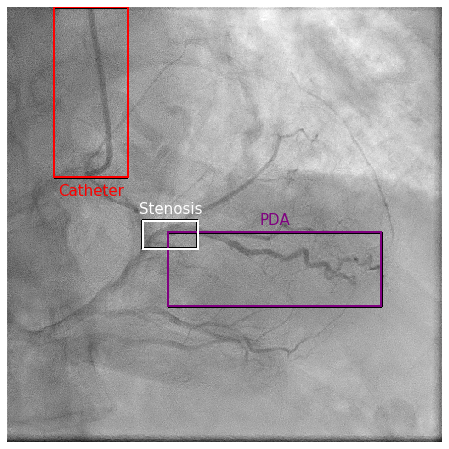  **Output**: PDA  (No Posterolateral bounding boxes were detected, and the stenosis box overlaps primarily with a PDA box.) | 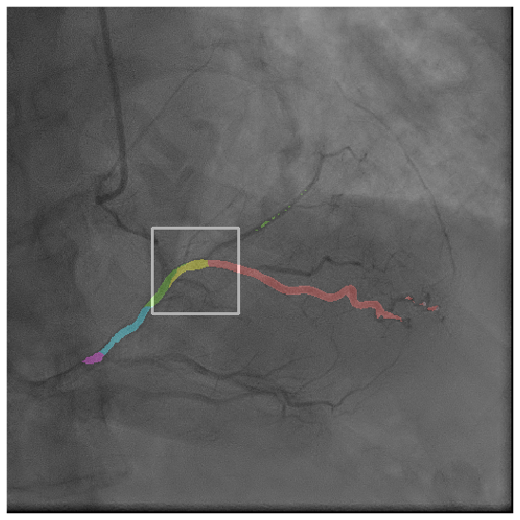  **Output**: Prox RCA  (Although Posterolateral pixels were detected within the resized stenosis box, the central pixels to which the stenosis was associated was Prox RCA.) |
| **LCA** | | |
| DeepCoro’s output and CathAI’s output are correct *(Ground truth: Leftmain)* | 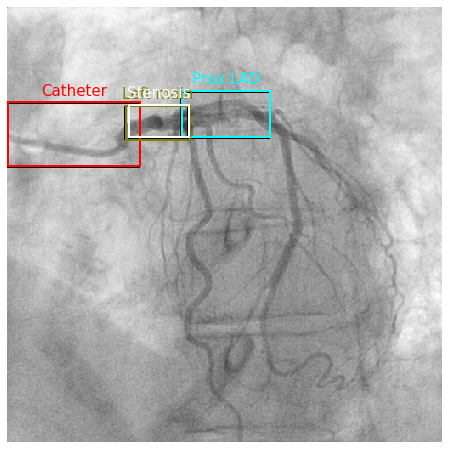  **Output**: Leftmain | 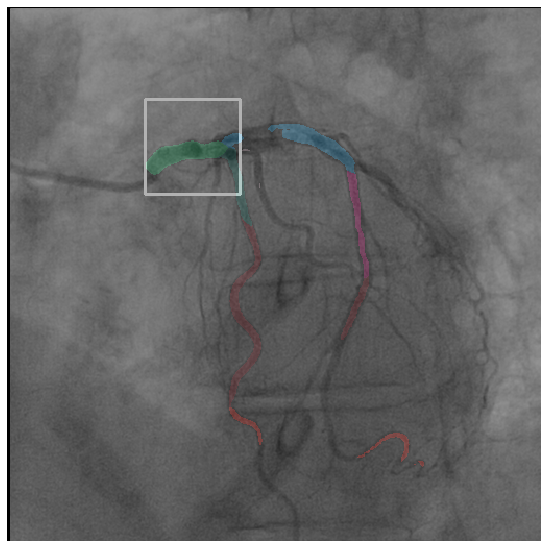  **Output**: Leftmain |
| DeepCoro’s output is correct and CathAI’s output is incorrect *(Ground truth: Prox LCX)* | 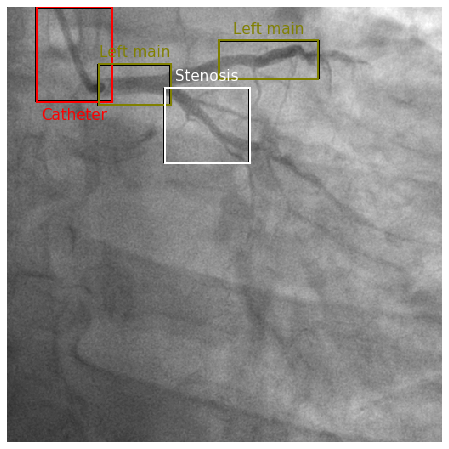  **Output**: Leftmain  (No Prox LCX bounding boxes were detected, and the stenosis box only overlaps with a Leftmain box.) | 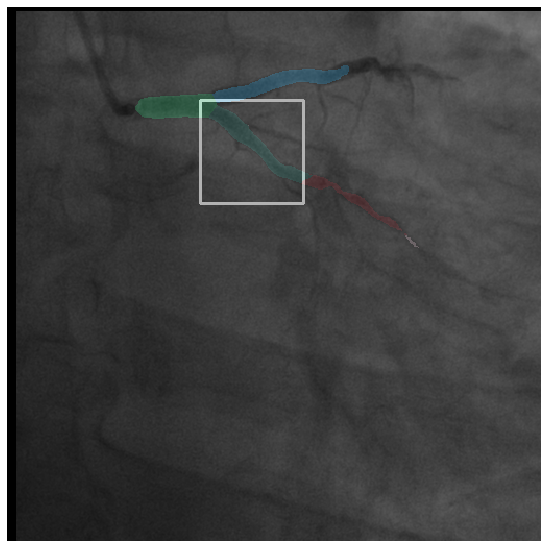  **Output**: Prox LCX |
| DeepCoro’s output is incorrect and CathAI’s output is correct *(Ground truth: Prox LCX)* | 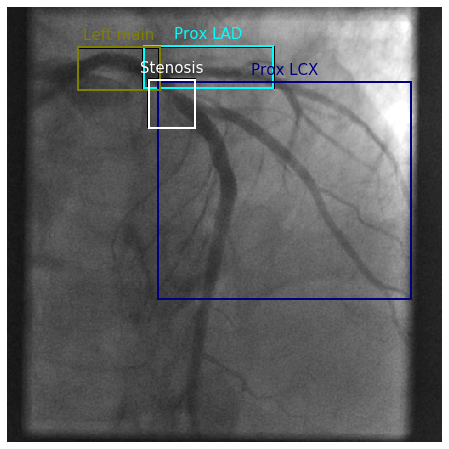  **Output**: Prox LCX | 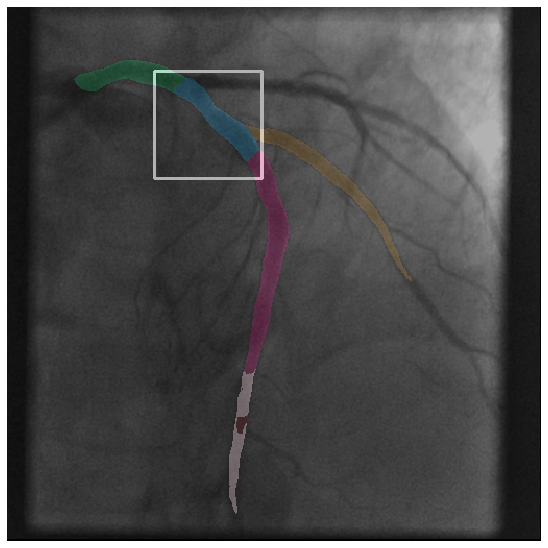  **Output**: Prox LAD  (No Prox LCX pixels were detected, and DeepCoro mistakenly identified the LAD segments of the LCA as the LCX segments.) |
| DeepCoro’s output and CathAI’s output are incorrect *(Ground truth: Dist LCX)* | 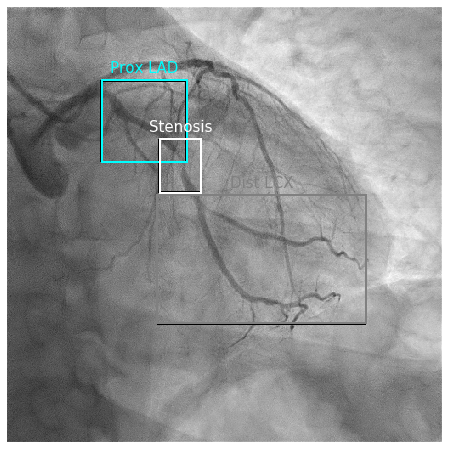  **Output**: Prox LAD  (CathAI mistakenly identified the LCX sub-segments and LAD sub-segments in the LCX branch of the LCA. The stenosis box only overlaps with Prox LAD.) | 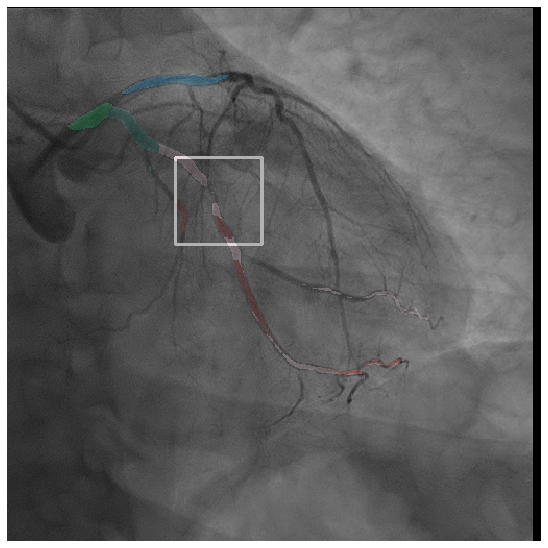  **Output**: Dist LAD  (No Dist LCX pixels were detected, and DeepCoro mistakenly identified the LCX sub-segments and LAD sub-segments in the LCX branch of the LCA.) |
| **Legends** | | |
|  |  |  |

**Legend**. Visual representation of results used to assign a coronary artery segment with CathAI and DeepCoro for four different cases. Unlike DeepCoro, which assesses the coronary artery tree as an interconnected structure, CathAI employs a method that identifies individual elements within an image using bounding boxes, without linking the various artery segments together. This approach results in CathAI assigning stenoses to specific bounding boxes without considering the underlying anatomy of the vessel. **Green highlight**: Correct output by the coronary artery segment assignment algorithm. **Red highlight**: Incorrect output by the coronary artery segment assignment algorithm. **Abbreviations**. Dist LAD: distal left anterior descending artery, Dist LCX: distal left circumflex artery, Dist RCA: distal right coronary artery, Left main: left main artery, LCA: Left Coronary Artery, Mid LAD: middle left anterior descending artery, Mid RCA: middle right coronary artery, PDA: posterior descending artery, Posterolateral: posterolateral branch from the right coronary artery, Prox LAD: proximal left anterior descending artery, Prox LCX, proximal left circumflex artery, Prox RCA: proximal right coronary artery, RCA: Right Coronary Artery.**Supplementary Table 13**. Performance of DeepCoro’s Algorithm 6 at the Video Level on Dataset A's Test Set, Segregated by According to the Number of Stenoses Associated to the Exam

| **Task** | **Metric** | **Coronary artery** | | | | | |
| --- | --- | --- | --- | --- | --- | --- | --- |
|  |  | **LCA** | | | | **RCA** | |
|  |  | **All** | | **LCX** | **LAD** |  |  |
|  |  | **Single stenosis** | **Multiple stenosis** | **Multiple stenosis** | **Multiple stenosis** | **Single stenosis** | **Multiple stenosis** |
| Number of videos | | 591 | 2,667 | 567 | 1,305 | 1,097 | 2,006 |
| Number of severe stenoses | | 52 | 724 | 191 | 451 | 102 | 475 |
| Classification | AUROC | 0.8493 (0.8259 - 0.8754) | 0.7542 (0.7447 - 0.7634) | 0.7270 (0.7063 - 0.7483) | 0.7576 (0.7454 - 0.7689) | 0.8988 (0.8839 - 0.9166) | 0.7999 (0.7878 - 0.8129) |
|  | AUPRC | 0.4405 (0.3616 - 0.5025) | 0.5394 (0.5207 - 0.5587) | 0.5745 (0.5365 - 0.6133) | 0.6112 (0.5878 - 0.6354) | 0.5968 (0.5441 - 0.6479) | 0.5913 (0.5670 - 0.6156) |
|  | Sensitivity (%) | 63.34 (57.14 - 70.01) | 67.42 (65.75 - 69.11) | 63.87 (60.66 - 67.31) | 69.84 (67.74 - 71.86) | 86.21 (83.33 - 89.61) | 75.37 (73.42 - 77.45) |
|  | Specificity (%) | 82.58 (80.92 - 84.30) | 69.58 (68.61 - 70.54) | 66.68 (64.38 - 69.08) | 66.32 (64.76 - 67.75) | 80.88 (79.63 - 82.12) | 70.75 (69.58 - 71.83) |
|  | PPV (%) | 25.99 (22.00 - 29.70) | 45.25 (43.87 - 46.62) | 49.35 (46.28 - 52.24) | 52.28 (50.21 - 54.19) | 31.60 (28.69 - 34.11) | 44.42 (42.81 - 46.08) |
|  | F1-score (%) | 36.83 (31.94 - 41.03) | 54.15 (52.89 - 55.46) | 55.67 (52.78 - 58.29) | 59.79 (58.06 - 61.47) | 46.23 (42.90 - 49.17) | 55.89 (54.25 - 57.52) |
| Regression | MAE (%) | 17.64 (17.06 - 18.17) | 24.72 (24.36 - 25.06) | 27.21 (26.33 - 28.04) | 25.89 (25.34 - 26.42) | 16.06 (15.64 - 16.48) | 20.69 (20.27 - 21.11) |
|  | r | 0.4385 (0.3816 - 0.4832) | 0.4325 (0.4157 - 0.4500) | 0.3911 (0.3529 - 0.4276) | 0.4607 (0.4399 - 0.4823) | 0.6126 (0.5813 - 0.6407) | 0.5539 (0.5346 - 0.5737) |

**Legend**. DeepCoro’s performance stratified across the number of stenoses. The range in parentheses is the 95% confidence interval generated by bootstrapping. **Abbreviations**. AUPRC: Area Under the Precision-Recall Curve, AUROC: Area Under the Receiver Operating Curve, LAD: Left Anterior Descending Artery, LCA: Left Coronary Artery, LCX: Left Circumflex Artery, MAE: Mean Absolute Error, PPV: Positive Predictive Value, r: Pearson’s correlation coefficient, RCA: Right Coronary Artery.

**Supplementary Table 14**. Time per DICOM to Analyse Every DICOM in the Test Set of Dataset A with DeepCoro from End-to-End

| **Part** | **The algorithm is applied to…** | **Time per DICOM (second) (mean ± SD)** |
| --- | --- | --- |
| Algorithm 1 | Every frame of the DICOM | 3.41 ± 0.72 |
| Algorithm 2 | Every frame of the DICOM | 8.73 ± 1.49 |
| Algorithm 3 | Every frame of the DICOM, in reference to each stenosis detected by Algorithm 2 | 24.31 ± 28.24 |
| Algorithm 4 | Every frame of the DICOM | 4.67 ± 2.14 |
| Algorithm 5 | Every registered segmented stenosis video | 0.84 ± 0.97 |
| Algorithm 6 | Every registered stenosis video kept | 10.12 ± 5.00 |
| Model loading and operations between algorithms | -- | 10.50 ± 2.38 |
| **Total** | | **62.60 ± 33.34** |

**Legend**. Average time and standard deviation for DeepCoro to analyse every DICOM in the test set of Dataset A, which have been separated per component of DeepCoro. Samples were analysed on a single NVIDIA RTX3090 GPU with a batch size of 1 for this analysis. **Abbreviations**. SD: Standard Deviation.

**Supplementary Table 15**. DeepCoro’s MAE in Videos from Dataset B Associated to Exam, PCI and CABG Procedures

| **Metric** | **Coronary artery** | | | | | | | | |
| --- | --- | --- | --- | --- | --- | --- | --- | --- | --- |
|  | ***LCA*** | | | ***RCA*** | | | ***RCA + LCA*** | | |
|  | ***Diagnostic Exam*** | ***PCI*** | ***CABG*** | ***Diagnostic Exam*** | ***PCI*** | ***CABG*** | ***Diagnostic Exam*** | ***PCI*** | ***CABG*** |
| Number of videos | 475 | 479 | 15 | 490 | 409 | 15 | 965 | 409 | 30 |
| Number of severe stenoses, ≥ 70% | 105 | 107 | 6 | 92 | 106 | 6 | 197 | 106 | 12 |
| MAE (%) | 20.26 (19.51 - 21.00) | 21.53 (20.70 - 22.37) | 21.60 (18.09 - 24.52) | 18.31 (17.56 - 18.98) | 21.29 (20.53 - 22.09) | 26.09 (21.41 - 30.61) | 19.27 (18.70 - 19.76) | 21.44 (20.83 - 21.98) | 23.86 (20.68 - 26.63) |

**Legend.** Difference in DeepCoro’s performance observed in the LCA and RCA for videos during the diagnostic coronary angiogram, during PCI (detected by the PCI detection algorithm) and in patients with previous CABG (detected by the CABG detection algorithm). Abbreviations. CABG: Coronary Artery Bypass Grafting, LCA: Left Coronary Artery, MAE: Mean Absolute Error, PCI: Percutaneous Coronary Intervention, RCA: Right Coronary Artery.

# Supplementary References

1 Avram, R. *et al.* CathAI: fully automated coronary angiography interpretation and stenosis estimation. *NPJ Digital Medicine* **6**, 142 (2023).

2 Sianos, G. *et al.* The SYNTAX Score: an angiographic tool grading the complexity of coronary artery disease. *EuroIntervention* **1**, 219-227 (2005).

3 Maxim Popov, A. A., Nuren Zhaksylyk, Alsabir Alkanov, Adilbek Saniyazbekov, Temirgali Aimyshev, Eldar Ismailov, Ablay Bulegenov, Alexey Kolesnikov, Aizhan Kulanbayeva, Arystan Kuzhukeyev, Orazbek Sakhov, Almat Kalzhanov, Nurzhan Temenov, & Siamac Fazli1. (ed Zenodo) (2023).
